# Metacognitive training for psychosis (MCT): a systematic meta-review of its effectiveness

**DOI:** 10.1038/s41398-025-03344-0

**Published:** 2025-04-22

**Authors:** Antonia Meinhart, Geneviève Sauvé, Annika Schmueser, Danielle Penney, Fabrice Berna, Łukasz Gawęda, Maria Lamarca, Steffen Moritz, Susana Ochoa, Caroline König, Vanessa Acuña, Rabea Fischer

**Affiliations:** 1 Department of Psychiatry and Psychotherapy, University Medical Center Hamburg, Hamburg, Germany; 2 Douglas Mental Health University Institute, Montréal, QC Canada; 3 Department of Education and Pedagogy, Université du Québec à Montréal, Montréal, QC Canada; 4 Department of Psychology, Université du Québec à Montréal, Montréal, QC Canada; 5 University of Strasbourg, University Hospital of Strasbourg, Inserm, Strasbourg, France; 6 Experimental Psychopathology Lab, Institute of Psychology, Polish Academy of Sciences, Warsaw, Poland; 7 Parc Sanitari Sant Joan de Déu, Sant Boi de Llobregat, Barcelona Spain; 8 Departament de Psicologia Clínica i de la Salut, Facultat de Psicologia, Universitat Autònoma de Barcelona, Bellaterra, Cerdanyola del Vallès, Barcelona Spain; 9 Consorcio de Investigación Biomédica en Red de Salud Mental (CIBERSAM), Instituto de Salud Carlos III, Madrid, Spain; 10 Grup MERITT, Fundació Sant Joan de Déu, Institut de Recerca Sant Joan de Déu, Esplugues de Llobregat, Barcelona Spain; 11 Soft Computing Research Group (SOCO) at Intelligent Data Science and Artificial Intelligence (IDEAI-UPC) Research Centre, Universitat Politecnica de Catalunya, UPC Barcelona Tech, Barcelona, Spain; 12 Departamento de Psiquiatría, Escuela de Medicina, Facultad de Medicina, Universidad de Valparaíso, Valparaíso, Chile; 13 Unidad de Trastornos Psicóticos, Hospital Del Salvador de Valparaíso, Valparaíso, Chile

**Keywords:** Schizophrenia, Scientific community

## Abstract

**Objective:**

Metacognitive training for psychosis (MCT) targets cognitive biases implicated in the pathogenesis of psychosis, e.g., jumping to conclusions, overconfidence in errors, and inflexibility. This systematic meta-review investigated the current meta-analytic evidence for the effectiveness of MCT with respect to core symptom features in schizophrenia (i.e., positive symptoms, delusions and hallucinations, negative symptoms, and overall psychotic symptoms).

**Data sources:**

This meta-review was registered with PROSPERO (CRD42023447442) on July 28, 2023. Articles were searched across five electronic databases from January 1, 2007 to September 1, 2023.

**Study selection:**

Meta-analyses addressing metacognitive interventions targeting psychotic symptoms were eligible for meta-review.

**Data extraction and synthesis:**

PRISMA guidelines were followed when applicable. Data extraction was done independently by two authors (AM, AS). A random-effects model was used to pool data within meta-analyses.

**Main outcomes and measures:**

Main outcomes were levels/severity of positive symptoms, delusions and hallucinations, negative symptoms, and overall psychotic symptoms after intervention.

**Results:**

Eight meta-analyses and two re-analyses were included for meta-review. A total of eight analyses provided sufficient data for analysis. Significant evidence was found in favor of MCT for positive symptoms (85.71%; *N* = 35, *g* = 0.473 [0.295, 0.651], *I*^*2*^ = 74.64), delusions (60%; *N* = 24, *g* = 0.639 [0.389, 0.889], *I*^*2*^ = 80.01), hallucinations (100%; *N* = 9, *g* = 0.265 [0.098, 0.432], *I*^*2*^ = 6.1), negative symptoms (100%; *N* = 17, *g* = 0.233 [0.1, 0.366], *I*^*2*^ = 34.78), and overall symptoms (50%; *N* = 37, *g* = 0.392 [0.245, 0.538], *I*^*2*^ = 65.73). None of the meta-analyses included a large enough sample size to meet the criteria for ‘suggestive’, ‘convincing’, or ‘highly convincing’ evidence according to *metaumbrella.org* guidelines (required sample size > 1000 cases). None of the meta-analyses scored ‘moderate’ or ‘high’ on methodological quality. Meta-analyses with significant results were more recent and/or considered more primary studies.

**Conclusions and relevance:**

There is consistent evidence that MCT ameliorates positive symptoms and delusions in schizophrenia.

## Introduction

Schizophrenia and related psychotic disorders have been ranked among the most debilitating mental disorders worldwide, requiring long-term disability adjustments for 1.5% of men and women aged between 25 and 49 years [1]. Moreover, schizophrenia has been linked to lifetime suicide rates as high as 10% [2], with rates of suicide attempts for people with schizophrenia ranging from 18–55% [3]. Guidelines from the American Psychiatric Association (APA), as well as the British NICE guidelines, recommend the use of antipsychotic drugs as first-line treatment for schizophrenia and psychosis [2]. In the past, psychotherapy for psychosis was often not offered due to the belief that the condition is neither psychologically explicable nor treatable [4]. Recent reviews with predominantly positive results [5, 6] have contradicted this viewpoint and discussed the inclusion of Cognitive Behavioral Therapy (CBT) in the treatment guidelines for psychosis. Yet, some dissenting findings have been reported [7], and effects of psychotherapy for psychosis are lower than for other disorders.

CBT targets symptoms by highlighting maladaptive beliefs or maladaptive appraisal and dysfunctional coping with events or situations [8–10]. A new trend in CBT is especially concerned with cognitive biases, such as jumping to conclusions (JTC), overconfidence in errors, and a bias against disconfirmatory evidence (BADE), which have been linked to the formation and maintenance of positive and negative symptoms [11–14] and are largely unresponsive to antipsychotic medication [15]. Metacognitive training for psychosis (MCT), a CBT variant, aims to raise (meta)cognitive awareness of these cognitive biases. The core principles and objectives of MCT relate to the concept of metacognition or the act of “thinking about thinking” [16]. MCT encompasses several aspects of self-awareness and problem-solving targeted at correcting cognitive distortions and overconfidence, as patients with schizophrenia spectrum disorders may not be aware of their cognitive biases or may tend to be overconfident in their assumptions [17–19] (see eTable 1 of the supplementary material for a complete overview of the MCT modules and objectives).

While an abundance of meta-analyses and reviews have investigated the effectiveness of CBT [5, 20, 21], less evidence is available for metacognitive approaches such as MCT. Several meta-analyses investigating the effectiveness, adherence, and feasibility of MCT have been published during the past decade, with the majority showing favorable results for MCT [22–26]; however, a few meta-analyses reported either unfavorable [27, 28] or inconclusive [29, 30] findings. To date, a critical meta-review addressing the methodological quality and robustness of these quantitative reviews is lacking. Meta-reviews provide a summary and grading of meta-analytic results and methodological quality in order to synthesize and identify the most credible evidence base for controversial or differentiated findings within the literature base [31]. Subsequently, meta-reviews may identify research domains in need of further evaluation as well as provide a direction for future research. Thus, the current study aimed to weigh the meta-analytic evidence on MCT’s effectiveness in reducing delusions as well as other positive, negative, and overall symptoms in patients with schizophrenia spectrum or related (non-affective) psychotic disorders.

## Methods

### Search strategy and selection criteria

This study was registered with PROSPERO (ID: CRD42023447442) on July 28, 2023. The following databases were searched between August 25 and September 1, 2023: PubMed, Web of Science, EMBASE, PsycINFO, and MEDLINE. The following search was conducted on PubMed: ((“Schizophrenia Spectrum and Other Psychotic Disorders”[Mesh]) OR (schizo* or delusion* or psychosis or psychoses or psychotic* or first episode* or first-episode* or FEP)) AND (((“metacognitive” train*) OR (“meta-cognitive” train*) OR (MCT)) AND (“2007”[Date - Publication] : “3000”[Date - Publication])) AND (*meta*-analys* OR metanalys* OR review). Similar searches were conducted in all databases (see eTable 2 in the supplementary material for a detailed description of the search strategy). We adhered to PRISMA guidelines, when applicable for this meta-review. Two authors (AM, AS) screened titles, abstracts, and full-text records independently. Any disagreements were resolved in discussion with two additional authors (RF, SM). Two search objectives were established. The first objective was to conduct a meta-review of MCT meta-analyses. The outcome of interest was defined as the severity of delusions after intervention compared to before intervention or to a different intervention/no treatment/treatment as usual (TAU)/active control. Secondary outcomes of interest included positive, negative, and overall psychotic symptoms. Outcomes at follow-up were also of interest. Due to the lack of studies investigating possible moderating effects of other baseline symptoms on the effectiveness of MCT for psychotic symptoms, particularly delusions, a second objective addressing moderating effects of baseline symptoms on the change in delusions only, as well as positive (i.e., delusions and hallucinations), negative, and overall symptoms was initially intended. The current study presents results for the first of the two objectives; the second objective was not further pursued in the framework of this article due to the aforementioned lack of studies.

Meta-analyses on metacognitive interventions were included for meta-review if they fulfilled the following inclusion and exclusion criteria: (i) samples comprised participants (mean age 18+ years) with a DSM/ICD diagnosis of a schizophrenia spectrum or related (non-affective) psychotic disorder, (ii) included interventions were group or individualized MCT+, which were either assessed separately or in combination against a control condition (no treatment, treatment as usual, active control), (iii) participants were compared before and after intervention or between conditions (no treatment, TAU, or active control, including treatments such as cognitive remediation, supportive therapy, and psychoeducation), (iv) included studies were meta-analyses that examined only MCT or psychological interventions targeting cognitive biases (i.e., metacognitive interventions) underlying delusions more broadly but included MCT as one of the interventions that measured clinical symptoms (including overall symptomatology, positive symptoms [i.e., delusions and hallucinations], negative symptoms) in people with psychosis. It has to be noted that results for effects on delusions, hallucinations, and positive symptoms (i.e., delusions and hallucinations) were separately evaluated for the purpose of this study.

Studies were excluded from the meta-review if they were conducted before 2007 (as the first MCT trial was published in 2007). Network meta-analyses were also excluded to minimize confounds pertaining to indirect evidence and inferences. Additionally, studies were excluded if they comprised participants with a diagnosis of affective psychosis or comprised samples made up of 60%+ of patients without a diagnosis of non-affective psychosis (as may be the case in studies recruiting participants with first-episode psychosis). Other literature to be excluded were single randomized controlled trials (RCTs), letters to the editor, study protocols, qualitative studies, case studies, editorial articles, book chapters, and systematic reviews if they were not coupled with a meta-analysis. Additionally, we excluded articles not written in English or German. Reference lists were searched for relevant studies, and experts in the field were contacted if necessary. See eTable 3, which provides a complete list and overview of all included articles, and eTable 4 which provides a list of the records and articles excluded based on the full text, both of which can be found in the supplementary material.

### Data analysis

The following data were extracted from the meta-analyses: meta-analysis authors, primary study authors, year of publication, effect size values, 95% confidence intervals for effect sizes, and sample sizes (total, cases, controls). Additionally, in cases with missing or incomplete data, the authors of the meta-analyses were contacted for further information. We needed to contact four authors [25, 26, 32, 33] for additional data and information, three of whom [25, 26, 32] provided datasets that were not available in the original meta-analyses. One meta-analysis [29] included only three primary studies, the data of which was too limited to be used for further statistical analysis. Another study [27] was excluded from further analysis due to multiple misclassifications and other methodological problems [10].

Study overlap, i.e., the extent to which the meta-analyses included the same primary studies, was assessed using a calculation of the corrected covered area (CCA) [34]. An assessment of study overlap is recommended when conducting meta-reviews [31] to critically evaluate the results of meta-analyses that have included and assessed most of the same primary studies. Study overlap can be categorized as slight (0–5%), moderate (6–10%), high (11–15%), and very high (>15%).

Methodological quality was independently assessed by two authors (AM, AS) using the revised A MeaSurement Tool to Assess systematic Reviews (AMSTAR-2) checklist [35] intended for meta-analyses that include randomized and non-randomized trials. Additionally, we used the adapted AMSTAR-Plus Content score [36]. The AMSTAR-2 is scored on 16 items and classifies study quality into four categories: high (no or one non-critical weakness), moderate (more than one non-critical weakness), low (one critical flaw with or without non-critical weaknesses), and critically low (more than one critical flaw with or without non-critical weaknesses). The AMSTAR-2 regards the following seven key components as critical items for assessment: pre-registered protocol, literature search adequacy, study exclusion justification, risk of bias for individual studies, appropriateness of meta-analytical methods, consideration of risk of bias in the discussion/interpretation of results, and assessment of likelihood and impact of publication bias. Correll et al.’s [36] AMSTAR-Plus Content scores range from 0 to 8 on the following items: double-blindness, total number of participants, significance of large study, observed cases, heterogeneity, and publication bias. See eTable 5 and eTable 6 in the supplementary material for full lists of the AMSTAR-2 and AMSTAR-Plus Content items.

Analyzable data were entered into metaumbrella.org, a statistical browser-based tool and its associated *R* package specifically designed for conducting meta-reviews [37]. To combine effect sizes from studies with different methods and sample characteristics, a random effects model was used. This model allows for the entered data to be converted into a common effect size [38] (Hedges’ *g*), and to provide a 95% confidence interval, as well as an assessment of heterogeneity using the *I*^2^ statistic [39]. Additionally, risk of bias, i.e., small study bias (smaller studies revealing more significant effects than larger studies), excess significance bias (excess of significant findings in the literature and publication bias), prediction intervals, and large study effects were assessed. Effect sizes were interpreted as ranging from small (*g* = 0.2) to medium (*g* = 0.5) to large (*g* = 0.8). A classification of evidence is provided, which can be divided into the following five categories: (i) convincing evidence (Class I: sample size >1000, *p*-value < 10e-6, *I*^2^ < 50%, *p*-value Egger’s test >0.05 and *p*-value Ioannidis test >0.05), (ii) highly suggestive evidence (Class II: sample size >1000, *p*-value < 10e-6, largest study with a statistically significant effect and Class I criteria not met), (iii) suggestive evidence (Class III: sample size >1000, *p*-value < 10e-3, Classes I–II criteria not met), (iv) weak evidence (Class IV: *p*-value < 0.05, Classes I–III criteria not met), and (v) non-significant (ns: *p*-value > 0.05). With the use of a classification and hierarchical grading of the available meta-analytic evidence, we aimed to provide a comparable and comprehensive overview of the more or less inconsistent findings regarding the effectiveness of MCT for psychotic symptom reduction.

### Role of the funding source

This project was supported by the Federal Ministry of Education and Research (BMBF), under the framework of ERA PerMed (ERAPERMED2022-292). The funding agency was not involved in the planning or execution of this study or in the evaluation of the results.

## Results

The initial data search yielded a total of 349 results, of which 199 duplicates were removed. After screening the articles based on title and abstract, 138 publications were excluded. After 12 records were screened based on full text, eight meta-analyses and two re-analyses were eventually included for meta-review. One study [29] provided unstandardized mean differences for three primary studies that were not convertible or analyzable for meta-review.

**Fig. 1:**
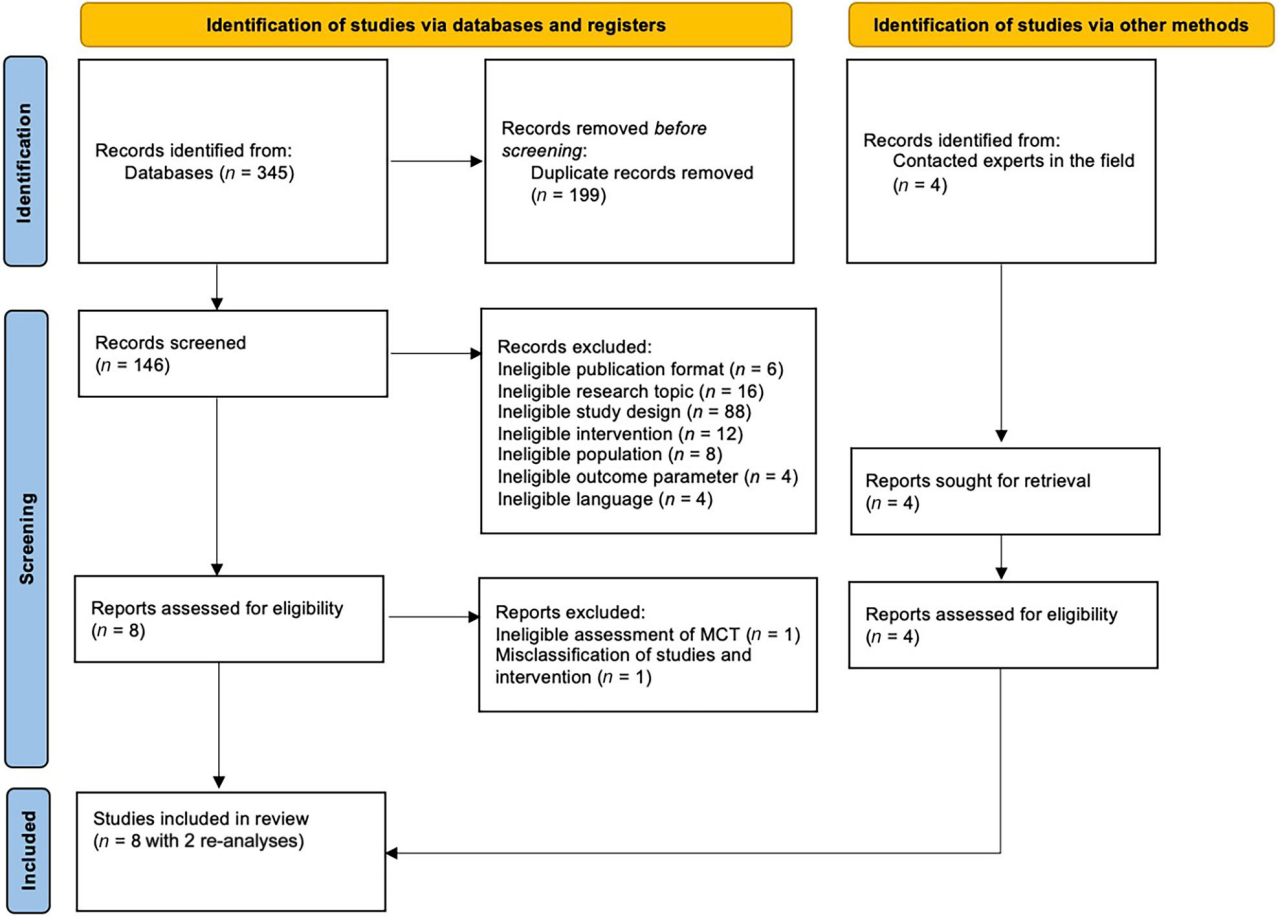
PRISMA flow diagram of the screening and selection process of records for this meta-review. MCT metacognitive training for psychosis.

After further assessment, one meta-analysis [27] was excluded from the analysis on the basis of extensive misclassification of interventions and imprecise administering of inclusion and exclusion criteria of primary studies [10]. Burlingame et al. [32] performed a re-analysis of their study [27], which was included in the meta-review and for which the original authors provided additional evidence upon request. Van Oosterhout et al. [28] were contacted but did not provide additional data for further analysis of their re-analysis [33]. Please refer to Fig. 1 for a flowchart of the screening and selection process. Additionally, Table 1 provides an overview of study characteristics for all studies included in the meta-review.

**Table 1:** Overview of study characteristics of included studies.

Meta-Analysis	Intervention	Control	Outcome parameters	*N* Studies (*n* Intervention; *n* Control)
Jiang et al. [30]	MCT	CogPack, ND, ST, TAU	Positive symptoms, delusions, global state, mental state, engagement with service, QoL, general functioning, adverse effects, dropout rate, satisfaction with treatment	11 (324; 322)
Eichner & Berna [22]	MCT	CogPack, ND, TAU, WL	Positive symptoms, delusions, subjective acceptance of the intervention	15 (408; 399)
^a^van Oosterhout et al. [28]	MCT, MCT+CBT, MSCT, MCT-JTC, MCT-T	CogPack, ST, TAU	Positive symptoms, delusions, data-gathering bias	11 (316; 617)
^b^van Oosterhout et al. [33]	MCT, MCT+, MCT+CBT, MCT-JTC, MCT-T	CogPack, ND, ST, TAU, WL	Positive symptoms, delusions, data-gathering bias	13 (375; 673)
Liu et al. [23]	MCT	CR+, ST, TAU, WL	Delusions	11 (352; 350)
Philipp et al. [24]	MCT	CogPack, HappyNeuron, health training, ND, ST, TAU, PE, PMR, WL	Positive symptoms	19 (597; 522)
Barnicot et al. [29]	MCT	CR, ND, TAU	General psychopathology, positive symptoms, relapse/re-hospitalization, social functioning, treatment compliance, JCT, delusions belief strength	3 (48; 46)
Sauvé et al. [25]	MCT, MCT+, MCTd, MCT-T, MCT+CACR, MCT+CR, MCT-JTC	CogPack, CR, ND, ST, TAU, WL	Positive symptoms, cognitive biases, insight	25 (606; 598)
^c^Burlingame et al. [32]	MCT	AC, AttCG, ST, TAU	Schizophrenia outcomes, group treatment-specific outcomes, general outcomes	11 (331; 315)
Penney et al. [26]	MCT, MCT+, MCT-JTC, MCT-ToM, MCT (virtual)	Community-based rehabilitation, current events discussion, CR, CR+, healthy living group, ND, PE, recreational activities, SS, ST, TAU, WL	Proximal outcomes (global positive symptoms, delusions, hallucinations, cognitive biases), distal outcomes (self-esteem, negative symptoms, QOL, well-being, social and global functioning)	43 (1272; 840)

*AC* active control, *AttCG* attention control group, *CR(+)* cognitive remediation therapy (individualized), *MCT(+)* metacognitive training (individualized), *MCTd* MCT for delusions, *MCT+CACR* MCT plus computer-assisted cognitive remediation, *MCT+CBT* MCT plus cognitive behavioral therapy, *MCT-JTC* MCT (target: jumping to conclusions), *MCT-T* MCT (targeted), *MCT-ToM* MCT (target: theory of mind), *MSCT* MCT plus social cognition training, *ND* newspaper discussion, *PE* psycho-education, *PMR* progressive muscle relaxation, *QoL* quality of life, *SS* social skills training, *ST* supportive therapy, *TAU* treatment as usual, *WL* waitlist.

^a^van Oosterhout et al.’s meta-analysis has been criticized for inconsistent application of inclusion/exclusion criteria and omitting eligible primary studies (see Moritz et al. [10]).

^b^van Oosterhout et al. [33] is a re-analysis of van Oosterhout et al.’s. [28] meta-analysis.

^c^Burlingame et al. [32] is a re-analysis of Burlingame et al.’s. [29] meta-analysis, which has been criticized for misclassification of interventions and studies (see Moritz et al. [10]).

### Study overlap

The overlap of primary studies included in the meta-analyses was calculated using the corrected covered area (CCA) [34]. Study overlap was assessed for all ten meta-analyses and across all outcome parameters, as well as for each outcome parameter (i.e., overall symptoms, positive symptoms, delusions, hallucinations, negative symptoms) separately (see Table 2). The total study overlap, calculated for all included meta-analyses and re-analyses as well as for all included primary studies assessing the effectiveness of MCT, was very high (22%). Study overlap for overall symptoms was slight (2%). While study overlap for positive symptoms (17.8%) and delusions (17.8%) was very high, the CCA for hallucinations (0%) and negative symptoms (0%) is none, which can be attributed to the fact that only one study (Penney et al. [26]) assessed hallucinations and negative symptoms as part of its outcome parameters.

**Table 2:** Corrected covered area (CCA; Pieper et al. [34]) for the assessment and classification of primary study overlap of the meta-analyses.

Variable	*k*	*r*	*c*	CCA	Classification
Overall symptoms	46	39	10	0.020	slight
Positive symptoms	117	45	10	0.178	very high
Delusions	65	24	10	0.178	very high
Hallucinations	9	9	10	0.000	none
Negative symptoms	17	17	10	0.000	none
Total	164	55	10	0.220	very high

Results were rounded to one-hundredth decimal.

*k* total number of included primary studies across meta-analyses, *r* number of index primary studies, *c* number of meta-analyses/re-analyses, *CCA* corrected covered area.

### Methodological quality (AMSTAR-2)

Table 3 and Table 4 depict the AMSTAR-2 assessments regarding critical and non-critical domains for each analyzable meta-analysis, respectively. Table 5 depicts the AMSTAR-Plus Content scores for each meta-analysis. Two re-analyses [32, 33] could not be assessed using the AMSTAR-2 and AMSTAR-Plus Content guidelines, as the provided information for methodological quality in these two articles was not sufficient to be conclusively scored on the relevant items of these checklists.

**Table 3:** Assessment of methodological quality of the meta-analyses regarding non-critical domains of the AMSTAR-2 checklist.

Meta-Analysis ID	PICO	Design	Screening	Extraction	Inclusion	Funding	Impact of RoB	Heterogeneity	Publication bias	Conflict of interest
Jiang [30]	yes	no	yes	yes	partial yes	no	yes	yes	no	yes
vanOosterhout [28]	yes	no	no	no	no	no	yes	yes	yes	yes
EichnerBerna [22]	yes	no	no	yes	no	no	yes	yes	yes	yes
Liu [23]	yes	no	yes	yes	partial yes	no	no	no	yes	no
Philipp [24]	yes	no	yes	yes	partial yes	no	yes	yes	yes	yes
Sauvé [25]	yes	no	yes	yes	partial yes	no	yes	yes	yes	yes
Penney [26]	yes	yes	yes	yes	partial yes	no	yes	yes	yes	yes

*AMSTAR-2* a measurement tool to assess systematic reviews (Shea et al. [35]), *PICO* population, intervention, comparator group, outcome, *RoB* risk of bias.

**Table 4:** Assessment of methodological quality of the meta-analyses regarding critical domains of the AMSTAR-2 checklist.

Meta-Analysis ID	Protocol	Search	Exclusion	Bias	Statistics	Discussion of bias	Publication bias
Jiang [30]	partial yes	partial yes	no	partial yes	yes	yes	no
vanOosterhout [28]	no	partial yes	yes	no	no	yes	yes
EichnerBerna [22]	no	partial yes	no	no	no	yes	yes
Liu [23]	no	partial yes	no	no	no	no	yes
Philipp [24]	yes	yes	yes	yes	no	yes	yes
Sauvé [25]	yes	partial yes	yes	partial yes	no	no	yes
Penney [26]	yes	yes	yes	partial yes	no	yes	yes

*AMSTAR-2* a measurement tool to assess systematic reviews (Shea et al. [35]).

**Table 5:** Assessment of methodological quality of the meta-analyses regarding AMSTAR-plus content scores.

Meta-Analysis ID	Double-blindness	Total *n*	Largest study sig.	Observed cases	Heterogeneity	Publication bias sig.	Total
Jiang [30]	0	1	0	1	0	0	2
vanOosterhout [28]	0	1	0	1	0	0	2
EichnerBerna [22]	0	1	0	1	0	0	2
Liu [23]	0	1	0	1	0	0	2
Philipp [24]	0	2	0	1	0	1	4
Sauvé [25]	0	2	0	1	1	0	4
Penney [26]	0	2	0	0	0	0	2

*AMSTAR-Plus Content* a measurement tool to assess systematic reviews plus content (Correll et al. [36]), *n* number of participants, *sig* significance.

All meta-analyses described the characteristics of PICO (population, intervention, comparator, outcome), three meta-analyses (42.85%) pre-registered a comprehensive protocol, two meta-analyses (28.57%) employed a comprehensive search strategy, four meta-analyses (57.14%) provided a list of and justifications for excluded studies, one meta-analysis (14.28%) comprehensively assessed risk of bias for the included primary studies, one meta-analysis (14.28%) employed appropriate statistical methods for combining results, five meta-analyses (71.42%) discussed risk of bias for the included primary studies when interpreting their results, and eight meta-analyses (85.71%) assessed publication bias. One meta-analysis provided a comprehensive explanation for inclusion of study design (14.28%), five meta-analyses (71.42%) performed study selection in duplicate, six meta-analyses (85.71%) performed data extraction in duplicate, none of the meta-analyses provided a detailed description of the included studies’ populations, none of the meta-analyses reported funding for the included primary studies, six meta-analyses (85.71%) assessed impact of included primary studies’ risk of bias on the results, six meta-analyses (85.71%) comprehensively discussed and explained heterogeneity in their results, and six meta-analyses (85.71%) appropriately reported conflicts of interest or funding for their study.

None of the meta-analyses had most primary studies of double-blind design, four meta-analyses (57.14%) had a total sample size *n* = 500–999, three meta-analyses (42.85%) had a total sample size of *n* > 1 000, none of the meta-analyses had results confirmed in an at least one- or two-arm large study with *n* > 200, one meta-analysis (14.28%) included observed cases in their study, two meta-analyses (14.28%) showed homogeneous results, and one meta-analysis (14.28%) did not show significant publication bias.

### Evidence classification

Table 6 provides an overview of the classification of the meta-analytic evidence. eFigs 1–10 of the supplementary material present all outcomes via forest plots (eFigs 1–5) and histograms (eFigs 6–10) by outcome parameter. We note that all calculations were performed using unweighted effect sizes. Thus, results may differ from the meta-analyses’ original results by a one-hundredth decimal. Additionally, two meta-analyses [22, 30] used weighted effect sizes in their original reports, whereas none of the effect sizes in the current meta-review have been weighted.

**Table 6:** Classification of meta-analytic evidence ranked by class of evidence, number of included primary studies, and date of publication^a^.

Outcome	Meta-Analysis ID	Intervention^b^/Control	RCT; nRCT	*n* cases	Hedges’ *g* [95% CI]^c^	*p*-value^d^	*I*^2^ (%)	PI 95% CI	SSE/ESB/LS	CE
Overall symptoms	Penney [26] [proximal]	MCT vs TAU (42.1%); MCT vs CR (10.52%); MCT vs ST (2.63%); MCT vs CBR (2.63%); MCT only (13.15%); MCT vs AC (15.78%); MCT vs PE (5.26%); MCT vs WL (7.89%)	25; 12	932	0.392 [0.245, 0.538]	0.000000162	65.73	[–0.368, 1.151]	no/no/no	IV
	Penney [26] [distal]	MCT vs TAU (38.46%); MCT vs CR (7.69%); MCT vs ST (3.84%); MCT vs CBR (3.84%); MCT vs PE (7.69%); MCT vs AC (15.38%); MCT only (19.23%); MCT vs WL (3.84%)	19; 7	645	0.313 [0.197, 0.43]	0.00000014	46.729	[–0.081, 0.707]	no/no/no	IV
	Burlingame [32]	MCT vs ND (20%); MCT vs TAU (20%); MCT vs CogPack (20%); MCT vs WL (20%); MCT vs PE (20%)	5; 0	162	0.142 [–0.08, 0.363]	0.209	0	[–0.218, 0.501]	no/no/no	ns
Positive symptoms	Penney [26]	MCT vs TAU (41.66%); MCT vs CogPack (8.33%); MCT vs CR (2.77%); MCT vs ST (2.77%); MCT vs CBR (2.77%); MCT only (13.88%); MCT vs OT (2.77%); MCT vs MCT + control (2.77%); MCT vs PE (5.55%); MCT vs WL (5.55%); MCT vs AC (8.33%)	23; 13	894	0.473 [0.295, 0.651]	0.00000019	74.635	[–0.483, 1.429]	no/no/no	IV
	Philipp [24]	MCT vs ND (10.05%); MCT vs CogPack (15.78%); MCT vs TAU (52.63%); MCT vs CR (5.26%); MCT vs ST (5.26%); MCT vs WL (5.26%); MCT vs PE (5.26%)	15; 4	531	0.302 [0.117, 0.486]	0.00135	49.248	[–0.317, 0.92]	no/no/no	IV
	Sauvé [25]	MCT vs ND (5.88%); MCT vs CogPack (17.64%); MCT vs TAU (41.17%); MCT vs CR (5.88%); MCT vs ST (5.88%); MCT only (5.88%); MCT vs MCT + control (5.88%); MCT+CR vs AC+WL (5.88%); MCT vs WL (5.88%)	9; 8	480	0.273 [0.099, 0.446]	0.00207	47.317	[–0.275, 0.821]	no/no/no	IV
	EichnerBerna [22]	MCT vs ND (9.09%); MCT vs TAU (45.45%); MCT vs ST (9.09%); MCT vs CogPack (18.18%); MCT vs WL (18.18%)	9; 2	245	0.361 [0.157, 0.565]	0.00052	7.127	[–0.023, 0.746]	no/no/no	IV
	Burlingame [32]	MCT vs ND (12.5%); MCT vs TAU (37.5%); MCT vs SS (12.5%); MCT vs CogPack (12.5%); MCT vs PE (12.5%); MCT vs group-based therapy (12.5%)	8; 0	241	0.19 [0.01, 0.37]	0.0385	0	[–0.035, 0.415]	no/no/no	IV
	Jiang [30]	MCT vs TAU (50%); MCT vs CogPack (25%); MCT vs ST (25%)	4; 0	129	0.406 [0.054, 0.758]	0.0238	34.301	[–0.808, 1.619]	yes/no/no	IV
	vanOosterhout [28,33]	MCT vs TAU (66.66%); MCT vs ST (11.11%); MCT vs CogPack (22.22%)	6; 3	209	0.26 [–0.003, 0.522]	0.0524	41.038	[–0.412, 0.931]	no/no/no	ns
Delusions	Penney [26]	MCT vs CogPack (13.04%); MCT vs TAU (56.52%); MCT vs CR (8.69%); MCT vs ST (4.34%); MCT vs CBR (4.34%); MCT only (8.69%); MCT vs AC (4.34%)	13; 9	621	0.639 [0.389, 0.889]	0.000000554	80.01	[–0.503, 1.781]	no/no/no	IV
	Liu [23]	MCT vs CogPack (27.27%); MCT vs ST (9.09%); MCT vs TAU (54.54%); MCT vs WL (9.09%)	11; 0	334	0.38 [0.125, 0.635]	0.00349	64.841	[–0.422, 1.183]	no/no/no	IV
	EichnerBerna [22]	MCT vs TAU (72.72%); MCT vs ST (9.09%); MCT vs CogPack (18.18%); MCT vs WL (9.09%)	9; 2	334	0.407 [0.066, 0.748]	0.0192	75.93	[–0.776, 1.59]	no/no/no	IV
	vanOosterhout [28,33]	MCT vs SC (14.28%); MCT vs TAU (57.14%); MCT vs CogPack (28.57%)	7; 0	224	0.234 [–0.035, 0.502]	0.0884	49.865	[–0.489, 0.956]	no/no/no	ns
	Jiang [30]	MCT vs TAU (50%); MCT vs CogPack (25%); MCT vs ST (25%)	4; 0	196	0.127 [–0.21, 0.464]	0.459	60.961	[–1.222, 1.476]	no/no/no	ns
Hallucinations	Penney [26]	MCT vs ST (11.11%); MCT vs CBR (11.11%); MCT vs TAU (33.33%); MCT only (22.22%); MCT vs CR (22.22%)	6; 3	271	0.265 [0.098, 0.432]	0.00185	6.108	[–0.095, 0.625]	no/no/no	IV
Negative symptoms	Penney [26]	MCT vs TAU (5.88%); MCT vs CR (11.76%); MCT vs CBR (5.88%); MCT vs TAU (29.41%); MCT vs AC (23.52%); MCT vs WL (5.88%); MCT only (11.76%); MCT vs RA (5.88%)	13; 4	415	0.233 [0.1, 0.366]	0.000592	34.777	[–0.125, 0.591]	no/no/no	IV

*RCT* randomized controlled trial, nRCT non-randomized controlled trial, *SSE* small study effect, *ESB* excess significance bias, *LS* largest study, *AttCG* attention control group, *TAU* treatment as usual, *AC* active control, *ND* newspaper discussion, *WL* waitlist, *PE* psychoeducation, *ST* supportive therapy, *CR* cognitive remediation therapy, *CBR* community-based rehabilitation, *RA* recreational activity, *OT* occupational therapy, *NA* not applicable / number of studies not sufficient for calculations.

^a^All calculations were performed using *metaumbrella.org*, a statistical tool, and its associated *R*-package designed for the use in meta-reviews; for all meta-analyses we used the REML/TESSPSST procedure. When calculating all meta-analyses with the latter setting, results converged and the level of significance remained unchanged.

^b^Metacognitive training (MCT) and all its adaptations are listed under the umbrella term MCT.

^c^All calculations were performed using unweighted effect sizes; thus, results may differ from the meta-analyses’ original results by a one-hundredth decimal. Meta-analyses that utilized weighted effect sizes include Jiang et al. [30] and Eichner and Berna [22].

^d^All *p*-values were rounded to the nearest one-hundredth decimal.

Significant evidence was found in favor of MCT for overall symptoms (50%; *N* = 37, *g* = 0.392 [0.245, 0.538], *I*^*2*^ = 65.73), positive symptoms (71.43%; *N* = 36, *g* = 0.473 [0.295, 0.651], *I*^*2*^ = 74.64), delusions (60%; *N* = 24, *g* = 0.639 [0.389, 0.889], *I*^*2*^ = 80.01), hallucinations (100%; *N* = 9, *g* = 0.265 [0.098, 0.432], *I*^*2*^ = 6.11), and negative symptoms (100%; *N* = 17, *g* = 0.233 [0.1, 0.366], *I*^*2*^ = 34.78). These effect sizes correspond to the study with the highest classification of evidence and largest sample size [26]; reporting of effect sizes has been adapted from Berendsen et al. [5]. Resulting effect sizes may slightly differ from the original published results, due rounding to the one-hundredth decimal of primary studies’ results and the application of a random-effects model used by *metaumbrella.org*. Meta-analyses with significant results were more recent and/or considered more primary studies.

#### Overall symptoms

One of the meta-analyses [26] that assessed overall symptoms reported significant, small to medium-sized effects for overall symptoms, specifically for proximal (*g* = 0.392, 95% CI [0.245, 0.538], *p* < 0.001, *I*^*2*^ = 65.73) and distal symptoms (*g* = 0.313, 95% CI [0.197, 0.43]), *p* < 0.001, *I*^*2*^ = 46.73) at end of treatment (EoT). The second meta-analysis [32] reported non-significant effect sizes for the effectiveness of MCT for overall symptoms of schizophrenia. Heterogeneity was considerable (*I*^2^ > 45%) for both significant effect sizes. None of the meta-analyses showed excess significance bias or small study effects, however the largest primary studies included were not significant.

#### Positive symptoms

Six of the seven (85.71%) meta-analyses [22, 24–26, 30, 32] that assessed positive symptoms reported significant, small to medium-sized effects (*g* = 0.473, 95% CI [0.295, 0.651], *p* < 0.001, *I*^*2*^ = 74.64; *g* = 0.302, 95% CI [0.117, 0.486], *p* < 0.005, *I*^*2*^ = 49.25; *g* = 0.273, 95% CI [0.099, 0.446], *p* < 0.005, *I*^*2*^ = 47.32; *g* = 0.361, 95% CI [0.157, 0.565], *p* < 0.005, *I*^*2*^ = 7.13; *g* = 0.19, 95% CI [0.01, 0.37], *p* < 0.05, *I*^*2*^ = 0; *g* = 0.406, 95% CI [0.054, 0.758], *p* < 0.05, *I*^*2*^ = 34.3) for the effectiveness of MCT for positive symptoms at EoT. Small study effects were detected for one meta-analysis [30] (16%). Nonetheless, all of these were ranked as ‘weak’ (Class IV) according to the grading criteria [37], which only allows a sample size of *n* experimental condition >1000 to be classified as Class III or higher. One meta-analysis [28] (14.28%) reported non-significant effect sizes. No excess significance bias was detected for any of the meta-analyses. None of the largest primary studies were significant.

#### Delusions

Three out of five (60%) meta-analyses [22, 23, 26] assessing delusions reported significant small to large-sized (*g* = 0.639, 95% CI [0.389, 0.889], *p* < 0.001, *I*^*2*^ = 80.01; *g* = 0.38, 95% CI [0.125, 0.635], *p* < 0.005, *I*^*2*^ = 64.84; *g* = 0.407, 95% CI [0.066, 0.748], *p* < 0.05, *I*^*2*^ = 75.93) effects for the effectiveness of MCT for delusions at EoT. Heterogeneity was considerable (*I*^2^ > 45%) for all three significant effect sizes. All evidence was ranked as ‘weak’ (Class IV) according to the grading criteria [37]. Two meta-analyses [28, 30] (40%) reported non-significant results. Neither small study effects, excess significance bias, nor the largest primary studies were significant for any of the meta-analyses.

#### Hallucinations

One meta-analysis [26] investigated the effectiveness of MCT for hallucinations, showing a significant, small to medium-sized effect in favor of MCT at EoT (*g* = 0.265, 95% CI [0.098, 0.432], *p* < 0.005, *I*^*2*^ = 6.1). The evidence was classified as ‘weak’ (Class IV). Neither small study effects nor excess significance bias was detected. The meta-analysis’ largest primary study [40] did not find significant effects for hallucinations.

#### Negative symptoms

One meta-analysis [26] investigated the effectiveness of MCT for negative symptoms. The results showed a significant but small effect in favor of MCT at EoT (*g* = 0.233, 95% CI [0.1, 0.366], *p* < 0.001, *I*^*2*^ = 34.77). The evidence was classed as ‘weak’ (Class IV). Neither small study effects nor excess significance bias was detected. The meta-analysis’ largest primary study [41] did not find significant effects for negative symptoms.

## Discussion

This meta-review aimed to provide a comprehensive overview of the meta-analytic evidence on the effectiveness of MCT in reducing the symptoms of schizophrenia, specifically delusions and positive symptoms. A total of eight meta-analyses and two re-analyses were considered for this meta-review.

Seven (87.5%; six meta-analyses and one re-analysis) out of eight statistically analyzable studies (seven meta-analyses (complemented by one re-analysis) and one re-analysis) provided evidence in favor of MCT at end of treatment (EoT), specifically for overall symptoms [26], positive symptoms [22, 24–26, 30, 32], delusions [22, 23, 26], hallucinations [26], and negative symptoms [26]. However, heterogeneity was substantial throughout all outcome measures. One study [32] reported no heterogeneity in their findings. Penney et al. [26] reported the highest amount of heterogeneity in their outcomes, while also encompassing the largest total sample size (*N* = 1816). Differences in heterogeneity could be ascribed several factors, including large sample sizes and increasing number of included studies, the variety of delivery formats of the intervention (e.g., in-person vs. online, number of sessions, in conjunction with other treatments vs. alone), as well as the setting (e.g., group vs. individual, inpatient vs outpatient setting) and staffing (e.g., nurse, psychologist) utilized during the treatment. Similar to other meta-reviews on interventions for schizophrenia symptoms (e.g., Berendsen et al. [5]), none of the meta-analytic evidence was classified as being ‘convincing’ (Class I) or ‘highly suggestive’ (Class II). All meta-analytic evidence was either classified as ‘weak (Class IV)’ or non-significant owing to an insufficient number of participants in the experimental conditions (*n* < 1 000) that is needed to be ranked Class III (‘suggestive’ evidence) or higher. None of the meta-analytic evidence regarding the effectiveness of CBT was ranked as ‘convincing’ (Class I) or ‘highly suggestive’ (Class II). Similar criteria have been used for the research and assessment of genetics (see Ioannidis et al. [42]) and dementia (see Bellou et al. [43]). Nonetheless, sample sizes for less prevalent disorders, such as psychosis and schizophrenia, are generally small (see Sauvé et al. [44]) and therefore less likely to fulfill the necessary classification criteria. Additionally, only 5–10% of treatments available in the medical literature show high levels of evidence, most of which (80%) are pharmacological interventions [45]. In combination with patients diagnosed with a psychotic disorder, who tend to show limited adherence to psychological interventions or treatments, these factors can considerably influence the observed effectiveness of metacognitive and other interventions in schizophrenia and psychosis. Thus, future studies should investigate patients’ satisfaction levels with MCT, which in turn may also influence adherence levels and treatment outcomes.

The most recent and broadest meta-analysis on the effectiveness of MCT to date was conducted by Penney et al. [26] and included 43 studies. The total sample size of that meta-analysis comprised 1816 participants and included 932 cases for proximal outcomes and 894 cases for positive symptoms. Penney et al. [26] found significant small (hallucinations, negative symptoms), medium-sized (proximal outcomes, distal outcomes, positive symptoms), and medium-to-large (delusions) effect sizes for all outcome variables of interest at EoT. All three meta-analyses reporting non-significant findings included less than ten primary studies [28, 30, 32].

Overlap of primary studies in our meta-review was very high for positive symptoms and delusions, suggesting that any discrepant results for meta-analyses addressing these outcomes might be due to methodological differences (i.e., effect size values, data analysis, and synthesis) rather than differences in samples and population. Total overlap for all meta-analyses and primary studies was accordingly classified as very high. Nonetheless, effects varied in size and even in status of significance, which is likely attributable to an increase in primary literature over time (e.g., differences in sample size and sample characteristics affecting results), methodology and data stratification. Hallucinations and negative symptoms were assessed by one meta-analysis [26]. Hence, overlap was none for these two outcomes.

Methodological quality of the meta-analyses was assessed using the AMSTAR-2 [35] and AMSTAR-Plus Content [36] checklists. Using these criteria, none of the meta-analyses were ranked as having high or moderate methodological quality. Two meta-analyses [24, 26] were ranked low, and all other meta-analyses received a critically low rank. The mean AMSTAR-Plus Content score was 2.44. None of the meta-analyses scored 5 or higher. Only two meta-analyses [24, 25] achieved a score of 4 (the maximum score can be achieved is 9). Additionally, double-blind primary studies were lacking, but this is inherently difficult to achieve in psychological intervention studies. While low scores indicate limited reliability of the data due to weaknesses in methodology, the AMSTAR-2 checklist [35] is subject to quite strict guidelines. Nonetheless, the authors state that “[…] our listing is a suggestion and appraisers may add or substitute other critical domains.” However, we decided to follow the suggested guidelines and a supplement to the AMSTAR-Plus Content [36] scores to ensure a comprehensive assessment.

### Strengths and Limitations

The main strengths of this meta-review are its systematic approach and the use of two assessment tools for the appraisal of methodological quality as well as a classification and overview of the analyzable evidence, with which we aimed to provide a critical assessment of the effectiveness of MCT for psychotic symptom reduction.

Nonetheless, we are also aware of some limitations to this meta-review. A comprehensive statistical analysis could not be conducted for two meta-analyses [29, 33] due to insufficient data. Moreover, none of the meta-analyses had a large enough sample size to be classified as Class III or higher (*n* cases > 1000). Any computations generated with metaumbrella.org [37] are subject to the use of a random-effects model. Also, we did not consider long-term studies in our meta-review because of a lack of relevant studies.

## Conclusion

The current study employed a systematic meta-review approach to investigate and evaluate the available meta-analytic evidence regarding the effectiveness of metacognitive training for psychosis (MCT) for reducing psychotic symptoms in schizophrenia spectrum or related, non-affective psychotic disorders. Seven out of eight analyzable studies (seven meta-analyses (one complemented by a re-analysis) and one re-analysis) provided evidence in terms of low-to-moderate effect sizes in favor of MCT at EoT and across all outcome parameters of interest. While MCT shows promising results for schizophrenia symptoms, showing the most favorable results for effects on delusions and positive symptoms, and represents a potentially cost-effective and easy-to-administer interventional tool, more studies with long-term outcomes and large sample sizes are needed for more powerful results, as well as more meta-analyses fulfilling AMSTAR-2 criteria.

### Data sharing

Supplementary data and results will be made available on our corresponding website at https://clinical-neuropsychology.de/metacognitive_training-psychosis/.

## Supplementary information

Supplementary Material
