# Supplementary material for: Metacognitive training for psychosis (MCT): a systematic meta-review of its effectiveness
Source: Transl Psychiatry. 2025 Apr 22;15:156. doi: 10.1038/s41398-025-03344-0 (PMC12015532; doi:10.1038/s41398-025-03344-0)

**Supplementary Material**

[eTable 1. Metacognitive Training for Psychosis Modules and Objectives 2](#_30j0zll)

[eTable 2. Search Strategy 4](#_1fob9te)

[eTable 3. List of Included Meta-Analyses 5](#_2et92p0)

[eTable 4. List of Excluded Articles 7](#_tyjcwt)

[eTable 5. AMSTAR-2 Items (based on Shea et al., 2017) 12](#_3dy6vkm)

[eTable 6. AMSTAR-Plus Content Items (Correll et al., 2017) 14](#_1t3h5sf)

[eFigure 1. Forest Plot Depicting Effect Sizes in Favor of MCT for Delusions 14](#_4d34og8)

[eFigure 2. Forest Plot Depicting Effect Sizes in Favor of MCT for Overall Symptoms 15](#_17dp8vu)

[eFigure 3. Forest Plot Depicting Effect Sizes in Favor of MCT for Positive Symptoms 15](#_3rdcrjn)

[eFigure 4. Forest Plot Depicting Effect Sizes in Favor of MCT for Hallucinations 16](#_26in1rg)

[eFigure 5. Forest Plot Depicting Effect Sizes in Favor of MCT for Negative Symptoms 16](#_lnxbz9)

[eFigure 6. Histogram Depicting Effect Sizes in Favor of MCT for Delusions 17](#_35nkun2)

[eFigure 8. Histogram Depicting Effect Sizes in Favor of MCT for Positive Symptoms 18](#_1ksv4uv)

[eFigure 9. Histogram Depicting Effect Sizes in Favor of MCT for Hallucinations 18](#_44sinio)

[eFigure 10. Histogram Depicting Effect Sizes in Favor of MCT for Negative Symptoms 19](#_2jxsxqh)

#

# eTable 1. Metacognitive Training for Psychosis Modules and Objectives

| Module | Target domain | Objective |
| --- | --- | --- |
| 1: Attribution | Attribution bias; monocausal inferences | Participants are trained to avoid succumbing to first impressions, which may eventually prove to be wrong (first task set) or only reveal half-truths (second task set). Things and situations can change over time, and increasing evidence often casts a different light on things. Therefore, alternative views and attitudes should not be dismissed prematurely. In our study, the pictures for the first task set did not elicit a jumping to conclusions pattern in schizophrenia patients (Moritz & Woodward, 2006b), but the exercises are well suited to demonstrating the disadvantages of such a response style. |
| 2: Jumping to conclusions I | Jumping to conclusions bias; bias against disconfirmatory evidence | As in Module 2 (Jumping to Conclusions I), explain to the group that it is often important to resist the normal tendency to stick to first impressions as this response bias fosters faulty decisions. It is therefore desirable to maintain an open mind. |
| 3: Changing beliefs | Bias against disconfirmatory evidence; jumping to conclusions bias | The main exercises consist of a series of three pictures shown in reverse order. The series of pictures gradually reveal an initially ambiguous plot. For each picture, ask participants to rate the plausibility of four different interpretations. The correct interpretation is highlighted at the end of each set of pictures. All exercises include at least one interpretation that is always unlikely no matter how many pictures have been presented. The examples are comprised of three different conditions that are presented in random order: revealed-on-first-picture, revealed-on-second-picture, and revealed-on-third-picture. |
| 4: To empathize I | Theory of mind; emotion perception | The first part of this module demonstrates that although facial expressions are very important for understanding the mental state and inner feelings of a person, they can also be misinterpreted quite easily. For instance, you cannot determine whether a person is an actor or a serial killer solely by examining their face. In order to adequately interpret a facial expression, it is important to consider other sources of information (e.g., context, personal background). Participants learn to consider a variety of contextual information rather than rely on single details. |
| 5: Memory | Overconfidence in errors | Although in most investigations patients with schizophrenia did not differ from controls on accuracy in the Deese-Roediger McDermott paradigm (Huron & Danion, 2002; for reviews, see Grimes & Zakzanis, 2018 or Moritz & Woodward, 2006a), their conviction regarding the accuracy of their memory errors was disproportionately high (Moritz, Woodward, et al., 2006). The present material elicits false memories in most people (i.e., irrespective of psychopathological status) and is instructive because it shows that even memories believed to be hard facts can be pseudo-memories. Participants should understand the fallibility of human memory. One of the objectives of this module is to teach participants to doubt their memory when they do not have a vivid recollection. In such cases, further proof is necessary, particularly for significant interpersonal situations (e.g., conflict). |
| 6: To empathize II | Complex theory of mind/social cognition; need for closure | The exercises demonstrate the difference between the patient’s level of information as an “omniscient viewer” and the facts available to the protagonists. For example, in one exercise in cycle A, a woman is given bad news by her doctor. When she arrives late for work, her boss scolds her. From the final picture, we cannot really tell whether her boss is cold-hearted or simply unaware of the doctor’s visit; it could be argued, however, that the boss should have acted more considerately since his employee likely appears devastated. Some of the comic scenes are unsatisfactory for persons with an increased need for closure. In many scenes - as in real life - definite explanations cannot be provided. Therefore, participants should propose what additional information is needed to ultimately verify one of the hypotheses. |
| 7: Jumping to conclusions II | Jumping to conclusions bias; liberal acceptance | Participants should learn that it is critical to invest sufficient time in finding the solution to complex problems. Sometimes, only additional information justifies clear-cut decisions, information that with only a superficial exploration would go unnoticed. |
| 8: Mood | Negative content schemata | Participants are introduced to dysfunctional thinking styles, which may contribute to the formation and maintenance of depression and low self-esteem. It should be emphasized that with regular training these cognitive styles can be corrected. |
| 9^a^: Self-esteem | Increasing self-esteem | Participants are made aware of how low self-esteem emerges. They learn not to focus on the negative aspects of their lives or their (supposed) personal flaws but to instead search for and appreciate the aspects of their life that are going well. Advice and suggestions for a daily routine are given, which may help strengthen participants’ self-esteem. |
| 10^a^: Dealing with prejudices (stigma) | (Self-)stigma | The module first discusses how common mental illnesses (such as psychosis) are in the general population. By giving examples of famous people, participants are shown that experiencing mental illness (such as psychosis) does not mean that one is unproductive or worthless. Participants are made aware of how stigmatization can influence their self-esteem. This module attempts to minimize participants’ self-stigma by increasing their awareness of the prevalence of mental illness in the general population. It is emphasized that mental illness, including psychosis, does not determine one’s worth, and participants are taught how to appropriately deal with their illness, such as by effectively communicating about their disorder to others. |

*Note*. This information has been adapted from the Metacognitive Training for Psychosis Manual (available free of charge at <https://clinical-neuropsychology.de/mct>).

^a^Modules 9 and 10 were considered ‘additional’ to the standard modules in MCT (sessions 1–8) at the time this meta-review was conducted and thus may not have been used in the metacognitive intervention analyzed in the included meta-analyses.

# eTable 2. Search Strategy

| Database | Search string | Date of search |
| --- | --- | --- |
| PubMed | (("Schizophrenia Spectrum and Other Psychotic Disorders"[Mesh]) OR (schizo* or delusion* or psychosis or psychoses or psychotic* or first episode* or first-episode* or fep))  AND ((("metacognitive" train*) OR ("meta-cognitive" train*) OR (MCT)) AND ("2007"[Date - Publication] : "3000"[Date - Publication])) AND (meta-analys* OR metanalys* OR review) | August 25, 2023 |
| PsycINFO | (psychosis/ or exp acute psychosis/ or exp affective psychosis/ or exp childhood psychosis/ or exp chronic psychosis/ or exp "paranoia (psychosis)"/ or exp schizophrenia/ or exp paranoid schizophrenia/ or (schizo* or delusion* or psychos* or psychotic* or first episode* or first-episode* or fep*).mp.) and (metacognitive train* or meta-cognitive train* or MCT).mp. and (meta-analys* or metaanalys* or review).mp. | August 25, 2023 |
| Web of Science | (((((((((ALL=((schizo* or delusion* OR psychosis OR psychoses OR psychotic* OR first episode* OR first-episode* OR fep*))) AND TS=((("metacognitive" NEAR train*) OR ("meta-cognitive" NEAR train*) OR (MCT)))) AND TS=((meta-analys* OR metaanalys* OR review)))) AND PY=(2007-2023)) OR (TS=((schizophrenia spectrum disorders))) OR ALL=(((schizo* or delusion* OR psychosis OR psychoses OR psychotic* OR first episode* OR first-episode* OR fep*)))) AND ALL=(((metacognitive train* OR meta-cognitive train* OR MCT)))) AND ALL=((meta-analys* OR metaanalys* OR review))) AND PY=(2007-2023)) | August 30, 2023 |
| EMBASE (Ovid) | (exp psychosis/ or exp acute psychosis/ or exp affective psychosis/ or exp brief psychotic disorder/ or exp childhood psychosis/ or exp delusion/ or exp depressive psychosis/ or exp endogenous psychosis/ or exp hallucination/ or exp intensive care psychosis/ or exp manic psychosis/ or exp paranoid psychosis/ or exp puerperal psychosis/ or exp schizophrenia/) OR ((schizo* or delusion* or psychos* or psychotic* or first episode* or first-episode* or fep*).mp.) AND ((metacognitive train* or meta-cognitive train*).mp. OR MCT.mp.) AND (meta-analys* OR metanalys* OR review).mp | August 30, 2023 |
| MEDLINE | (exp "schizophrenia spectrum and other psychotic disorders"/ OR (schizo* or delusion* or psychos* or psychotic* or first episode* or first-episode* or fep*).tw,kf.) AND ((("metacognitive" adj train*) or ("meta-cognitive" adj train*)).mp. OR MCT.tw,kf.) AND (meta-analys* OR metanalys* OR review).mp | September 1, 2023 |

#

# eTable 3. List of Included Meta-Analyses

| Meta-Analysis | doi | Search Strategy | | Funding / Financial Support |
| --- | --- | --- | --- | --- |
|  |  | Databases | Search Terms^a^ |  |
| Jiang et al., 2015 | 10.11919/j.issn.1002-0829.215065 | CENTRAL,  Current Contents, EMBASE, MEDLINE, PsycINFO, Web of Science | [‘randomized controlled trial’/exp OR ‘randomized controlled trial’ AND (metacogniti*:ab,ti OR (‘meta’ NEAR/2 (‘cognitive’ OR ‘cognition’)):ab,ti) AND schizophreni*:ab,ti] | This study was funded by the Science and Technology Commission of Shanghai Municipality (13z2260500); the Shanghai Municipal Commission of Health and Family Planning (2013ZYJB0020); the Shanghai Shen Kang Hospital Development Center (SHDC12014111); and the Shanghai Health System Leadership in Health Research Program (XBR2011005). |
| van Oosterhout et al., 2016a | 10.1017/s0033291715001105 | CENTRAL, Cochrane Database of Systematic Reviews, EMBASE, MEDLINE, PsycINFO | ‘metacognitive training’ OR ‘MCT’) AND outcome research (search terms included ‘randomised controlled trial’, ‘randomized controlled trial’ OR ‘RCT’ | *Not disclosed.* |
| van Oosterhout et al., 2016b | 10.1017/s003329171600009x | Not provided/re-analysis | Not provided/re-analysis | *Not disclosed.* |
| Eichner & Berna, 2016 | 10.1093/schbul/sbv225 | CENTRAL, EMBASE, PsycINFO, PubMed | (delusion* or psychosis or psychotic or schizophren*) and (metacogn* or reason* or cognitive bias*) and (training or therap* or intervention) | This study did not receive financial support. |
| Liu et al., 2018 | 10.1111/wvn.12282 | CINAHL, Cochrane Library, Joanna Briggs Institute Library, MEDLINE, PsycINFO | delusion (psychosis or psychotic or schizophrenia) and metacognitive (training or therapy or intervention) | *Not disclosed.* |
| Philipp et al., 2019 | 10.1002/cpp.2345 | BIOSIS, CINAHL, ClinicalTrials.gov,  GoogleScholar, ICRTP, ISI Web of Science, MEDLINE, Open Grey, ProQuest Dissertations, PsycINFO | (metacogniti* or “meta cogniti*” or “MCT”); metacognition AND (randomized OR effectiveness) AND (disorder OR illness) AND (therapy OR treatment) +MCT +mental -child -adolescent | This work was funded by the German Federal Ministry of Education and Research (grant number 01KG1511). |
| Barnicot et al., 2020 | 10.1016/j.cpr.2020.101929 | EMBASE, MEDLINE, PsycInfo | (“inpatient” or “hosp*”), (“psychiatr*” or “mental”), (“psycho*” or “therap*” or “train*” or “group*” or “interven*”) | *Not disclosed.* |
| Sauvé et al., 2020 | 10.1016/j.cpr.2020.101854 | EMBASE, MEDLINE, PsycINFO | (schizophreni* OR psychosis OR psychoses OR psychotic*) AND (cogniti* OR think* OR reason*) AND (bias* OR error* OR distort* OR style) | This research was not funded by a specific granting agency, or commercial or/not-for-profit sector. Salary awards include: doctoral award from the Fonds de Recherche du Québec – Santé (FRQeS) for author GS; postdoctoral fellowship from the Canadian Institutes of Health Research (CIHR) for author KML; FRQ-S Research Scholar salary award for author MBB; and James McGill Professorship from McGill University and Research Chair from the FRQ-S for author ML. The funding sources had no role in the study design, collection, analysis, or interpretation of the data, writing the manuscript, or the decision to submit the paper for publication. |
| Burlingame et al., 2022 | 10.1037/pst0000433 | Not provided/re-analysis | Not provided/re-analysis | *Not disclosed.* |
| Penney et al., 2022 | 10.1001/jamapsychiatry.2022.0277 | CINAHL (EBSCO), Cochrane Central Register of Controlled Trials, EMBASE (Ovid), MEDLINE (Ovid), OpenGrey, ProQuest Dissertations, PsycINFO (Ovid), PubMed, Social Science Research Network eLibrary, Social Work Abstracts (Ovid), Web of Science | (schizo* or delusion* or psychosis or psychoses or psychotic* or first episode* or first-episode* or fep*) TX All Text AND (metacognitive train* or meta-cognitive train*) TI Title  OR (metacognitive train* or meta-cognitive train*) AB Abstract OR (metacognitive train* or meta-cognitive train*) TX All Text; (("Schizophrenia Spectrum and Other Psychotic Disorders"[Mesh]) OR (schizo* or delusion* or psychosis or psychoses or psychotic* or first episode* or first-episode* or fep)) AND ((("metacognitive" train*) OR ("meta-cognitive" train*) OR (MCT)) AND ("2007"[Date - Publication] : "3000"[Date - Publication])) | Funding for this research was provided by the Canada First Research Excellence Fund, awarded through the Healthy Brains, Healthy Lives (HBHL) initiative at McGill University (grant HBHL 3c-KM-56). Dr Lepage was supported by a James McGill Professorship from McGill University. Dr Thibaudeau was supported by a postdoctoral fellowship from the Canadian Institutes of Health Research (171198). The funding organizations had no role in the design and conduct of the study; collection, management, analysis, and interpretation of the data; preparation, review, or approval of the manuscript; or decision to submit the manuscript for publication. |

*Note*. ^a^The provided search strategies and search terms listed here are similar for each of the databases searched for each meta-analysis.

# eTable 4. List of Excluded Articles

| Article | doi | Reason for exclusion | Comment |
| --- | --- | --- | --- |
| Aerts et al., 2010 | 10.1111/j.1600-6143.2009.02883.x | Ineligible research topic | Chylous Ascites Requiring Surgical Intervention after Donor Nephrectomy: Case Series and Single Center Experience |
| Ahuir et al., 2018 | 10.1016/j.psychres.2018.10.066 | Ineligible study design | Randomized crossover clinical trial |
| Alvarez-Astorga et al., 2019 | 10.1016/j.pnpbp.2019.109672 | Ineligible study design | Clinical trial |
| Andreou et al., 2017 | 10.1016/j.jbtep.2016.11.013 | Ineligible study design | Randomized controlled rater-blind study |
| Atmaca, 2022 | 10.5152/alphapsychiatry.2022.22840 | Ineligible population | Obsessive-compulsive disorder |
| Balzan et al., 2014 | 10.1177/0004867413508451 | Ineligible study design | Clinical trial |
| Balzan et al., 2019 | 10.1093/schbul/sby152 | Ineligible study design | Randomized clinical trial |
| Barbieri et al., 2020 | 10.1708/3333.33020 | Publication language other than English or German | Italian |
| Barros et al., 2014 | 10.1590/1519-6984.04013 | Ineligible research topic | Possible impacts of climate change on wetlands and its biota in the Brazilian Amazon |
| Batmaz et al., 2021 | 10.5498/wjp.v11.i9.589 | Ineligible study design | Review article; no meta-analysis for MCT |
| Bhardwaj et al., 2017 | 10.2500/ar.2017.8.0202 | Ineligible research topic | Effect of topical beclomethasone on inflammatory markers in adults with eosinophilic esophagitis |
| Bighelli et al., 2018 | 10.1002/wps.20577 | Ineligible study design | Network meta-analysis |
| Bonnin et al., 2014 | 10.1097/HRP.0000000000000062 | Ineligible research topic | Restoring functioning in euthymic bipolar disorder via functional remediation |
| Borsboom et al., 2016 | 10.1017/S0033291715001944 | Ineligible study design | Review article; no meta-analysis for MCT |
| Brar et al., 2020 | 10.1093/schbul/sbaa031.257 | Ineligible study design | Scoping review; no meta-analysis for MCT |
| Brar et al., 2022 | 10.1080/17522439.2021.1918753 | Ineligible study design | Scoping review; no meta-analysis for MCT |
| Briki et al., 2014 | 10.1016/j.schres.2014.06.005 | Ineligible study design | Randomized controlled trial |
| Brüne et al., 2013 | 10.1024/1661-4747/a000165 | Ineligible study design | Review article; no meta-analysis for MCT |
| Burlingame et al., 2020 | 10.1037/pst0000293 | Misclassification of studies and intervention | Misclassification of interventions, misclassification of studies, inconsistent application of inclusion/exclusion criteria |
| Caponnetto et al., 2018 | 10.4081/mi.2018.7812 | Ineligible study design | Randomized, controlled, monocentric, single-blind trial |
| Cella et al., 2015 | 10.3389/fpsyg.2015.01259 | Ineligible study design | Review article; no meta-analysis for MCT |
| Chan & Mak, 2012 | 10.1016/j.cpr.2012.06.001 | Ineligible study design | Review article; no meta-analysis for MCT |
| Churchill et al., 2010 | 10.1002/14651858.CD008705 | Ineligible population | Depression; study protocol |
| Cupo et al., 2021 | 10.1093/schbul/sbaa183 | Ineligible outcome parameter | Nonpsychotic and subthreshold psychotic symptoms prior to emergence of a first episode of psychosis |
| Darmedru et al., 2018 | 10.1016/j.encep.2017.05.001 | Ineligible intervention | Cognitive remediation |
| da Veiga et al., 2012 | 10.1002/jmv.23198 | Ineligible research topic | Viral load and epidemiological profile of patients infected by pandemic influenza A (H1N1) 2009 and seasonal influenza A virus in Southern Brazil |
| de Assis da Silva et al., 2020 | 10.1590/0047-208500000046 | Publication language other than English or German | Portuguese |
| de Pinho et al., 2020 | 10.1111/jan.14240 | Ineligible study design | Randomized controlled trial; study protocol |
| Dietrichkeit et al., 2017 | 10.1159/000464340 | Ineligible study design | Review article; no meta-analysis for MCT |
| Dietrichkeit et al., 2020 | 10.1024/1661-4747/a000415 | Ineligible study design | Review article; no meta-analysis for MCT |
| Dimaggio & Lysaker, 2010 | ISBN 1136999981, 9781136999987 | Ineligible publication format | Book |
| Enriquez-Barreto & Morales, 2016 | 10.1186/s40591-016-0047-9 | Ineligible research topic | The PI3K signaling pathway as a pharmacological target in Autism related disorders and schizophrenia |
| Favrod et al., 2011 | 10.1111/j.1365-2648.2010.05470.x | Ineligible study design | Uncontrolled pilot study |
| Favrod et al., 2014 | 10.1016/j.eurpsy.2013.08.003 | Ineligible study design | Randomized controlled trial |
| Fekete et al., 2022 | 10.1111/sjop.12811 | Ineligible study design | Single-blind randomized controlled trial |
| Fekete et al., 2023 | 10.1007/s12144-022-02794-9 | Ineligible study design | Clinical trial |
| Fekete et al., 2016 | PMID: 27852968 | Publication language other than English or German | Hungarian |
| Fieker & Schneider, 2015 | 10.1007/s00278-015-0003-1 | Ineligible study design | Review article; no meta-analysis for MCT |
| Fischer et al., 2022 | 10.1002/cpp.2755 | Ineligible study design | Case study |
| Garnham, 2015 | 10.1080/17470218.2015.1029950 | Ineligible publication format | Book review |
| Gawęda et al., 2009 | PMID: 20209879 | Ineligible study design | Review article; no meta-analysis for MCT |
| Gawęda et al., 2009 | PMID: 20209880 | Ineligible study design | Review article; no meta-analysis for MCT |
| González-Blanch et al., 2021 | 10.1016/j.jpsychires.2021.06.040 | Ineligible study design | Randomized controlled trial |
| Gorini da Veiga et al., 2012 | 10.1002/jmv.23198 | Ineligible research topic | Viral load and epidemiological profile of patients infected by  pandemic influenza A (H1N1) 2009 and seasonal influenza A virus in Southern Brazil |
| Gregersen et al., 2022 | 10.1093/schbul/sbac060 | Ineligible study design | Follow-up cohort study |
| Gussmann et al., 2023 | 10.3389/fpsyt.2023.1160075 | Ineligible study design | Intervention mapping |
| Han & Lee, 2022 | 10.1016/j.apnu.2022.07.010 | Ineligible study design | Pretest-posttest quasi-experimental study |
| Hasson-Ohayon et al., 2009 | PMID: 19827697 | Ineligible study design | Review article; no meta-analysis for MCT |
| Haugen et al., 2022 | 10.1016/j.scog.2022.100275 | Ineligible study design | Clinical trial |
| Hauke et al., 2022 | 10.1093/schbul/sbac029 | Ineligible study design | Clinical trial |
| Hesse & Klingberg, 2018 | 10.1055/a-0645-0704 | Ineligible intervention | Cognitive behavioral therapy |
| Hua & Kanas, 2020 | 10.1080/00207284.2019.1704630 | Ineligible study design | Review article; no meta-analysis for MCT |
| Hunot et al., 2010 | 10.1002/14651858.CD008704 | Ineligible population | Depression |
| Ishikawa et al., 2020 | 10.1016/j.schres.2019.08.006 | Ineligible study design | Randomized controlled trial |
| Iyer et al., 2021 | 10.1177/0706743720961714 | Ineligible study design | Open-label single-blind randomized controlled trial |
| Jelinek et al., 2019 | 10.1111/bjc.12213 | Ineligible study design | Randomized controlled trial |
| Jelinek et al., 2017 | 10.1038/s41598-017-03626-8 | Ineligible population | Depression |
| Jelinek et al., 2018 | 10.1024/1016-264X/a000211 | Ineligible population | Obsessive-compulsive disorder |
| Jensen et al., 2021 | 10.1164/rccm.202105-1150OC | Ineligible research topic | Association between intermittent hypoxemia and severe bronchopulmonary dysplasia in preterm infants |
| Knowles et al., 2016 | 10.1002/jclp.22312 | Ineligible intervention | Attention training technique |
| Kolbeck et al., 2017 | 10.1007/s00278-017-0240-6 | Ineligible study design | Review article; no meta-analysis for MCT |
| Korman et al., 2020 | 10.1016/j.psychres.2019.112601 | Ineligible intervention | High intensity interval training |
| Kubota & Riek, 2022 | 10.1146/annurev-control-042920-093225 | Ineligible population | Methods for robot behavior adaptation for cognitive neurorehabilitation |
| Kühne et al., 2017 | 10.1136/bmjopen-2016-015428 | Ineligible study design | Systematic review protocol |
| Larkin & Hutton, 2017 | 10.1192/bjp.bp.116.193458 | Ineligible outcome parameter | Treatment decision-making capacity in psychosis |
| Leanza et al., 2020 | 10.1016/j.jbtep.2020.101547 | Ineligible study design | Randomized rater-blind controlled trial |
| Lee et al., 2019 | 10.1016/B978-0-12-815312-3.00007-3 | Ineligible research topic | Military-to-civilian transition process for ill/injured service members |
| Lopez-Morinigo et al., 2020 | 10.1017/S0033291720003384 | Ineligible outcome parameter | Insight |
| Lopez-Morinigo et al., 2020 | 10.1186/s12888-020-2431-x | Ineligible study design | Randomized controlled trial; study protocol |
| Lopez-Morinigo et al., 2021 | 10.2196/26548 | Ineligible intervention | Use of ecological momentary assessment through a passive smartphone-based app (eB2) by patients with schizophrenia: Acceptability study |
| Lux et al., 2020 | 10.1007/s00115-020-00916-x | Ineligible study design | Review article; no meta-analysis for MCT |
| Lüdtke et al., 2023 | 10.1016/j.jbtep.2023.101885 | Ineligible study design | Narrative review article; no meta-analysis for MCT |
| Lysaker et al., 2013 | 10.2478/s13380-013-0131-4 | Ineligible study design | Review article; no meta-analysis for MCT |
| Lysaker et al., 2013 | 10.2217/fnl.12.78 | Ineligible study design | Review article; no meta-analysis for MCT |
| Lysaker et al., 2018 | 10.2147/PRBM.S146446 | Ineligible study design | Review article; no meta-analysis for MCT |
| Makowski et al., 2020 | 10.1017/S0033291719002071 | Ineligible intervention | Intersection of verbal memory and expressivity on cortical contrast and thickness in first episode psychosis |
| Manoli et al., 2021 | 10.1037/prj0000481 | Ineligible outcome parameter | Vocational outcomes |
| Martiadis et al., 2023 | 10.3389/fpsyt.2023.1155321 | Ineligible study design | Review article; no meta-analysis for MCT |
| Mehl et al., 2023 | 10.1007/s00115-022-01433-9 | Ineligible study design | Narrative review article |
| Mendelson et al., 2022 | 10.1016/j.scog.2021.100230 | Ineligible study design | Clinical trial |
| Menon et al., 2017 | 10.3371/CSRP.MEBA.022015 | Ineligible study design | Review article; no meta-analysis for MCT |
| Morales Corado et al., 2022 | PMID: 35862567 | Ineligible research topic | Carnitine-acylcarnitine translocase deficiency |
| Moritz et al., 2010 | 10.1097/YCO.0b013e32833d16a8 | Ineligible study design | Review article; no meta-analysis for MCT |
| Moritz et al., 2011 | 10.1017/S0033291710002618 | Ineligible study design | Randomized controlled trial |
| Moritz et al., 2014 | 10.1016/j.cpr.2014.04.004 | Ineligible study design | Narrative review article; no meta-analysis for MCT |
| Moritz et al., 2015 | 10.3389/fpsyg.2015.01048 | Ineligible study design | Clinical trial |
| Moritz, 2016 | 10.1017/S0033291716000192 | Ineligible study design | Comment |
| Moritz et al., 2016 | 10.1017/S0033291715002068 | Ineligible study design | Comment |
| Moritz et al., 2016 | 10.1586/14737175.2016.1135737 | Ineligible study design | Review article; no meta-analysis for MCT |
| Moritz et al., 2017 | 10.1037/abn0000262 | Ineligible study design | Empirical study |
| Moritz et al., 2017 | 10.1159/000464256 | Ineligible study design | Review article; no meta-analysis for MCT |
| Moritz et al., 2017 | 10.1016/j.jbtep.2016.07.004 | Ineligible study design | Review article; no meta-analysis for MCT |
| Moritz et al., 2018 | 10.1016/j.eurpsy.2018.05.010 | Ineligible study design | Randomized controlled trial |
| Moritz et al., 2019 | 10.31887/DCNS.2019.21.3/smoritz | Ineligible study design | Review article; no meta-analysis for MCT |
| Moritz & Lysaker, 2018 | 10.1016/j.schres.2018.06.001 | Ineligible study design | Review article; no meta-analysis for MCT |
| Moritz & Woodward, 2007 | 10.1097/YCO.0b013e3282f0b8ed | Ineligible study design | Review article; no meta-analysis for MCT |
| Moritz et al., 2023 | 10.1007/s00406-022-01394-9 | Ineligible study design | Review article; no meta-analysis for MCT |
| Müller, 2019 | 10.17192/z2019.0325 | Ineligible research topic | Quality of the parent-child relationship and course of symptoms of attention deficit hyperactivity disorder (ADHD) in kindergarten age |
| Palumbo et al., 2018 | Website link: https://old.jpsychopathol.it/article/the-implementation-of-cognitive-remediation-interventions-in-campania/ | Ineligible intervention | Cognitive remediation |
| Palumbo et al., 2019 | 10.2147/NDT.S221690 | Ineligible intervention | CIRCuiTS (computerized cognitive remediation therapy program) |
| Palumbo et al., 2022 | 10.3389/fpsyt.2022.833550 | Ineligible study design | Randomized controlled trial; study protocol |
| Pankowski et al., 2016 | 10.12740/PP/59113 | Ineligible study design | Narrative systematic review article; no meta-analysis for MCT |
| Paquin et al., 2023 | 10.1037/abn0000806 | Ineligible study design | Longitudinal study |
| Parellada, 2013 | 10.1176/appi.ajp.2013.13081041 | Ineligible publication format | Book |
| Parikh et al., 2015 | 10.1111/bdi.12233 | Ineligible population | Bipolar disorder |
| Park et al., 2020 | 10.1017/S1352465819000560 | Ineligible study design | Randomized controlled trial |
| Péneau & Franck, 2015 | 10.1016/j.amp.2015.02.002 | Publication language other than English or German | French |
| Philipp et al., 2020 | 10.1007/s10942-019-00333-3 | Ineligible study design | Review article; no meta-analysis for MCT |
| Pillny & Lincoln, 2020 | 10.1007/s00115-019-00831-w | Ineligible intervention | Cognitive behavioral therapy |
| Pontillo et al., 2017 | 10.3390/brainsci7100127 | Ineligible intervention | Did not include metacognitive training |
| Pöttgen, 2018 | 10.1024/1016-264X/a000214 | Ineligible population | Multiple sclerosis |
| Rakitzi et al., 2020 | 10.1027/1016-9040/a000400 | Ineligible study design | Systematic review article; no meta-analysis for MCT |
| Reeder et al., 2017 | 10.1017/S0033291717001234 | Ineligible study design | Randomized controlled trial |
| Renou & Doyen, 2019 | 10.1016/j.amp.2018.07.012 | Ineligible research topic | NEAR (neuropsychological educational approach to cognitive remediation) cognitive remediation program in adolescents with attention deficit/hyperactivity disorder and/or autism spectrum disorder |
| Roberts & Penn, 2013 | ISBN 0199777586, 9780199777587 | Ineligible publication format | Book |
| Rouy et al., 2022 | 10.1037/xge0001185 | Ineligible study design | Conceptual replication study |
| Rüegg et al., 2018 | 10.1024/1016-264X/a000213 | Ineligible study design | Pilot study; uncontrolled trial |
| Ruiz-Delgado et al., 2022 | 10.1016/j.psychres.2022.114941 | Ineligible study design | Randomized controlled trial |
| Saheki & Song, 2005 | PMID: 20301360 | Ineligible research topic | Citrin deficiency |
| Samochowiec et al., 2021 | 10.12740/PP/OnlineFirst/135527 | Ineligible study design | Consensus statement |
| Schaich et al., 2018 | 10.3389/fpsyt.2018.00584 | Ineligible study design | Single-center randomized clinical trial |
| Schaub et al., 2010 | ISBN 9780203855782 | Ineligible publication format | Book chapter |
| Schneider et al., 2016 | 10.1186/s12888-016-0756-2 | Ineligible study design | Randomized controlled trial |
| Schneider et al., 2018 | 10.1027/2151-2604/a000336 | Ineligible study design | One-armed open-label intervention study |
| Scorza et al., 2009 | 10.1016/j.plefa.2009.06.008 | Ineligible research topic | Omega-3 consumption and sudden cardiac death in schizophrenia |
| Shan et al., 2021 | 10.1007/s00406-020-01119-w | Ineligible study design | Clinical trial |
| Sharef et al., 2013 | 10.5001/omj.2013.101 | Ineligible research topic | Successful treatment of cardiomyopathy due to very long-chain acyl-CoA dehydrogenase deficiency: First case report from Oman with literature review |
| Sharma et al., 2022 | 10.7759/cureus.23424 | Ineligible study design | Narrative review; no meta-analysis for MCT |
| Siddiqui et al., 2019 | 10.1016/j.schres.2018.10.012 | Ineligible intervention | Multitasking in the City Test |
| Solmi et al., 2023 | 10.1038/s41380-022-01727-z | Ineligible study design | Network meta-analysis |
| Storebø et al., 2011 | 10.1002/14651858.CD008223.pub2 | Ineligible research topic | Social skills training for attention deficit hyperactivity disorder (ADHD) in children aged 5 to 18 years |
| Szczepanowski et al., 2020 | 10.3389/fpsyt.2020.00725 | Ineligible study design | Empirical study |
| Tai & Turkington, 2009 | 10.1093/schbul/sbp080 | Ineligible study design | Review article; no meta-analysis for MCT |
| Tanoue et al., 2020 | 10.1111/jjns.12389 | Ineligible study design | Prospective, multicenter, single-group pre-post study |
| van der Gaag et al., 2013 | ISBN: 1135052700, 9781135052706 | Ineligible publication format | Book |
| Veckenstedt et al., 2021 | 10.30820/2364-1517-2021-2-149 | Ineligible study design | Practical introduction to metacognitive training+ |
| Vianin, 2016 | 10.3389/fpsyt.2016.00056 | Ineligible intervention | Cognitive remediation |
| Wang et al., 2022 | 10.3390/brainsci12030413 | Ineligible study design | Randomized controlled trial |
| Ward & Garety, 2019 | 10.1016/j.schres.2017.08.045 | Ineligible study design | Review article; no meta-analysis for MCT |
| Wood et al., 2020 | 10.1016/j.schres.2020.03.041 | Ineligible assessment of Metacognitive Training | Did not provide a (subgroup) analysis for metacognitive training |
| Zhuang et al., 2011 | 10.1016/j.proeng.2011.11.010 | Ineligible research topic | Formulation and physicochemical characterisation of a novel self-microemulsifying delivery system as hydrotropic and solubilising agent for penfluridol |

# eTable 5. AMSTAR-2 Items (based on Shea et al., 2017)

| Item | Criteria |
| --- | --- |
| 1: Did the research questions and inclusion criteria for the review include the components of PICO (Population, Intervention, Control, Outcome)? | Describes population, intervention, control, outcome |
| 2: Did the report of the review contain an explicit statement that the review methods were established prior to the conduct of the review and did the report justify any significant deviations from the protocol? | The authors state that they had a written protocol or guide that included ALL the following: review question(s), search strategy, inclusion/exclusion criteria, risk of bias assessment; as for partial yes, plus the protocol should be registered and should also have specified: meta-analysis/synthesis plan *and* plan for investigating causes of heterogeneity, justifications for any deviations from the protocol |
| 3: Did the review authors explain their selection of the study designs for inclusion in the review? | The review should satisfy ONE of the following: explanation for including only RCTs OR explanation for including only NSRI OR explanation for including both, RCTs and NRSI |
| 4: Did the review authors use a comprehensive literature search strategy? | Searched at least two databases (relevant to research question), provided keyword and/or search strategy, justified publication restrictions (e.g., language); searched the reference lists/bibliographies of included studies, searched trial/study registries, included/consulted content experts in the field, searched for grey literature, conducted search within 24 months of completion of the review |
| 5: Did the review authors perform study selection in duplicate? | At least two reviewers independently agreed on selection of eligible studies and achieved consensus on which studies to include OR two reviewers selected a sample of eligible studies and achieved good agreement (at least 80 per cent), with the remainder selected by one reviewer |
| 6: Did the review authors perform data extraction in duplicate? | At least two reviewers achieved consensus on which data to extract from included studies OR two reviewers extracted data from a sample of eligible studies and achieved good agreement (at least 80 percent), with the remainder extracted by one reviewer |
| 7: Did the review authors provide a list of excluded studies and justify the exclusions? | Provided a list of all potentially relevant studies that were read in full text form but excluded from the review; justified the exclusion from the review of each potentially relevant study |
| 8: Did the review authors describe the included studies in adequate detail? | Described populations, interventions, comparators, outcomes, research designs; described population in detail, described intervention and comparator in detail (including doses where relevant), described study’s setting, timeframe for follow-up |
| 9: Did the review authors use a satisfactory technique for assessing the risk of bias (RoB) in individual studies that were included in the review? | RCTs: Unconcealed allocation, and lack of blinding of patients and assessors when assessing outcomes (unnecessary for objective outcomes such as all cause mortality); allocation sequence that was not truly random, and selection of the reported result from among multiple measurements or analyses of a specified outcome  NRSI: From confounding, and from selection bias; methods used to ascertain exposures and outcomes, and selection of the reported result from among multiple measurements or analyses of a specified outcome |
| 10: Did the review authors report on the sources of funding for the studies included in the review? | Must have reported on the sources of funding for individual studies included in the review. Note: Reporting that the reviewers looked for this information but it was not reported by study authors also qualifies |
| 11: If meta-analysis was performed, did the review authors use appropriate methods for statistical combination of results? | RCTs: The authors justified combining the data in a meta-analysis AND they used an appropriate weighted technique to combine study results and adjusted for heterogeneity if present AND investigated the causes of any heterogeneity  NRSI: The authors justified combining the data in a meta-analysis AND they used an appropriate weighted technique to combine study results, adjusting for heterogeneity if present AND they statistically combined effect estimates from NRSI that were adjusted for confounding, rather than combining raw data, or justified combining raw data when adjusted effect estimates were not available AND they reported separate summary estimates for RCTs and NRSI separately when both were included in the review |
| 12: If meta-analysis was performed, did the review authors assess the potential impact of RoB in individual studies on the results of the meta-analysis or other evidence synthesis? | Included only low risk of bias RCTs OR, if the pooled estimate was based on RCTs and/or NRSI at variable RoB, the authors performed analyses to investigate possible impact of RoB on summary estimates of effect |
| 13: Did the review authors account for RoB in individual studies when interpreting/discussing the results of the review? | Included only low risk of bias RCTs OR, if RCTs with moderate or high RoB, or NRSI were included the review provided a discussion of the likely impact of RoB on the results |
| 14: Did the review authors provide a satisfactory explanation for, and discussion of, any heterogeneity observed in the results of the review? | There was no significant heterogeneity in the results OR if heterogeneity was present the authors performed an investigation of sources of any heterogeneity in the results and discussed the impact of this on the results of the review |
| 15: If they performed quantitative synthesis, did the review authors carry out an adequate investigation of publication bias (small study bias) and discuss its likely impact on the results of the review? | Performed graphical or statistical tests for publication bias and discussed the likelihood and magnitude of impact of publication bias |
| 16: Did the review authors report any potential sources of conflict of interest, including any funding they received for conducting the review? | The authors reported no competing interests OR The authors described their funding sources and how they managed potential conflicts of interest |

# eTable 6. AMSTAR-Plus Content Items (Correll et al., 2017)

| Item | Criteria |
| --- | --- |
| 1: Was the majority of all meta-analyzed studies double-blind? | 0 points: Can’t answer/ Double-blind studies: < 84% of all included studies  1 point: Double-blind studies: 85–94% of all included studies  2 points: Double-blind studies: 95–100% of all included studies |
| 2: Was the total number of participants in the meta-analysis sufficiently large? | 0 points: Can’t answer / Total *n* < 500  1 point: Total *n* = 500–999  2 points: Total *n* >/= 1000 |
| 3: Was the meta-analytically derived primary outcome result confirmed in at least one large study with approximately 100 patients per arm? | 0 points: Can’t answer/Not confirmed in study with *n* >/= 200  1 point: Confirmed in a two-arm study with *n* >/= 200  2 points: Confirmed in a three-arm study with *n* >/= 300 |
| 4: Were studies with observed cases analyses included in the meta-analysis? | 0 points: Can’t answer/yes  1 point: No |
| 5: Was the primary outcome result heterogeneous? | 0 points: Can’t answer/yes  1 point: No (*Q* statistic = *p* > 0.05 and *I^2^* < 50%) |
| 6: Was there significant publication bias regarding the primary outcome result? | 0 points: Can’t answer/yes  1 point: No (Egger’s test *p* > 0.05, symmetrical funnel plot) |

# eFigure 1. Forest Plot Depicting Effect Sizes for Delusions


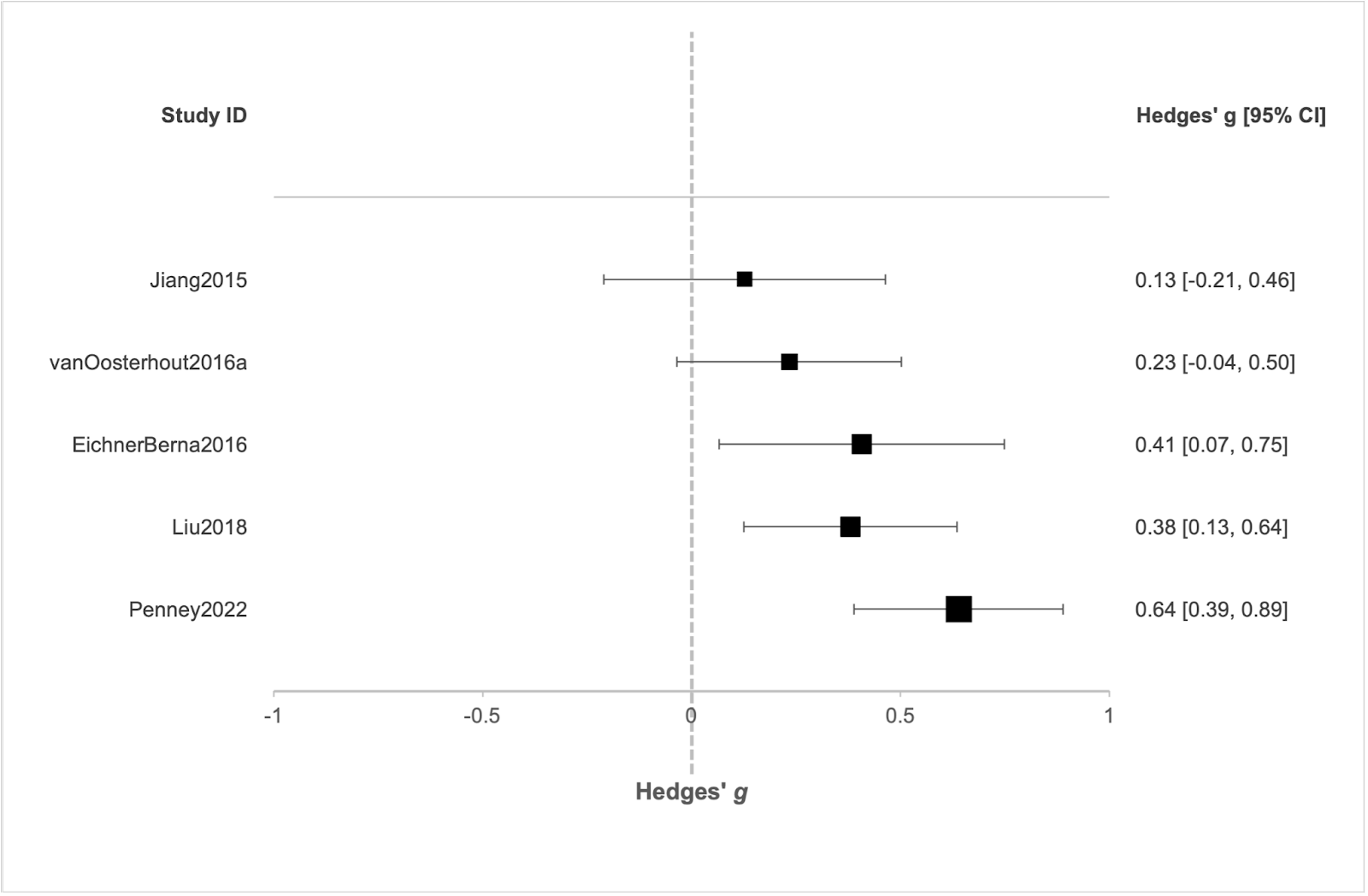


*Note*. Positive effect size values favor the intervention.

#

# eFigure 2. Forest Plot Depicting Effect Sizes for Overall Symptoms


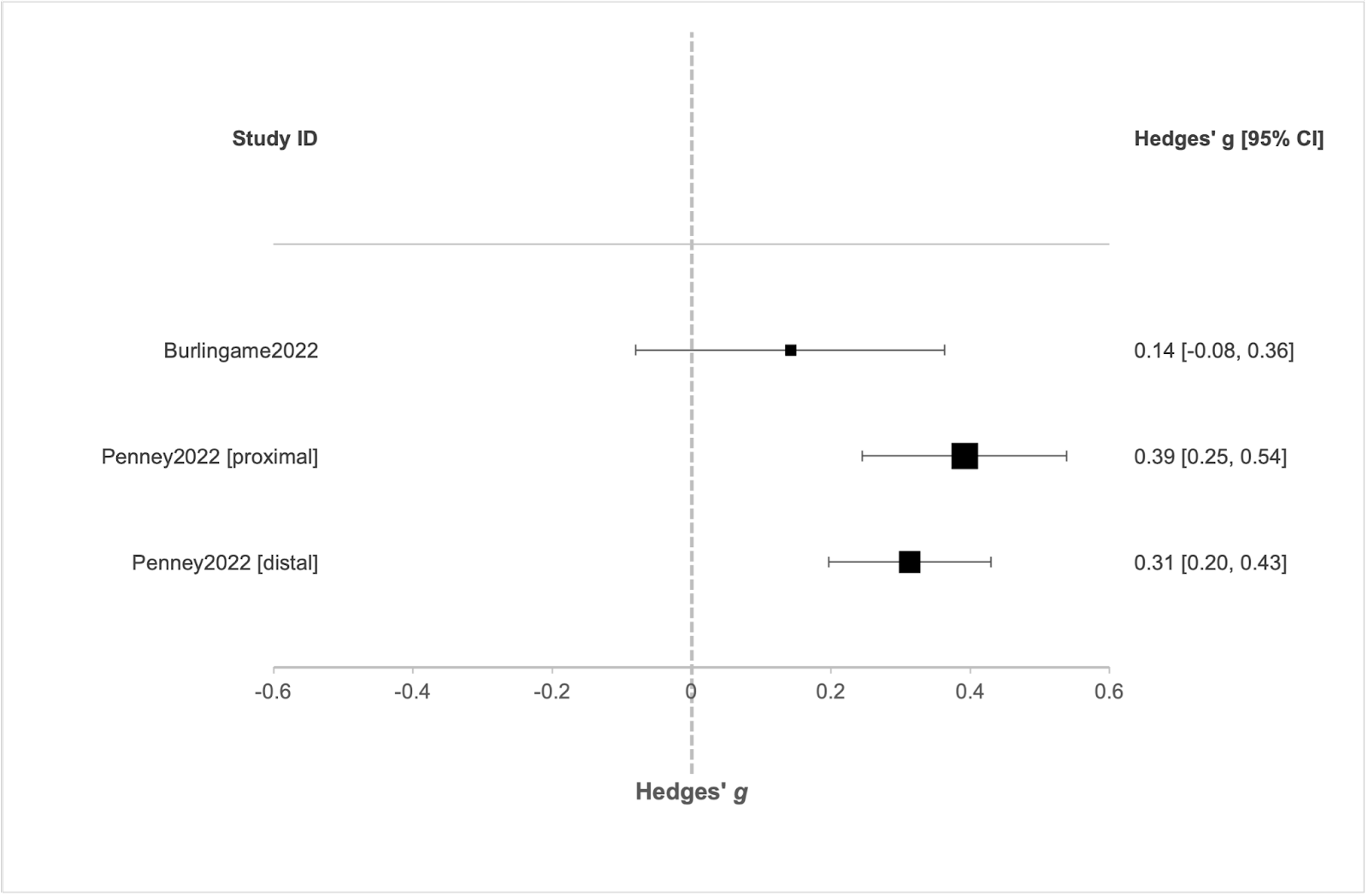


*Note*. Positive effect size values favor the intervention.

# eFigure 3. Forest Plot Depicting Effect Sizes for Positive Symptoms


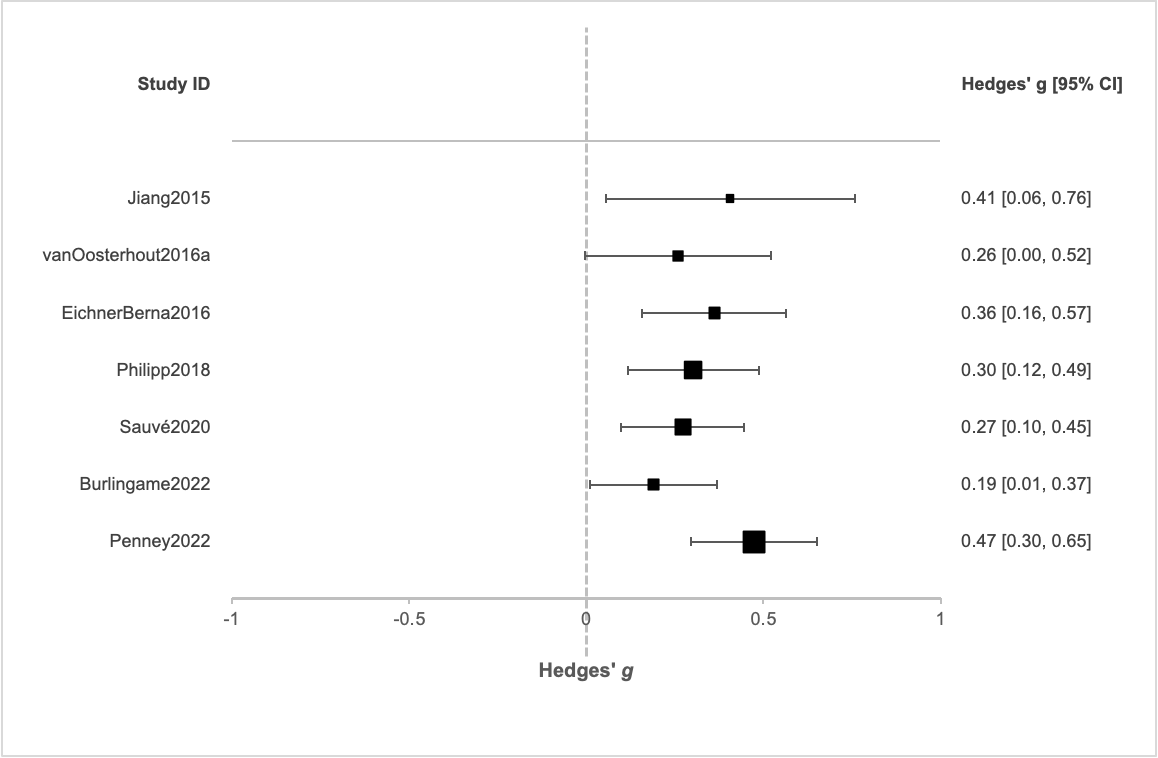


*Note*. Positive effect size values favor the intervention.

# eFigure 4. Forest Plot Depicting Effect Sizes for Hallucinations


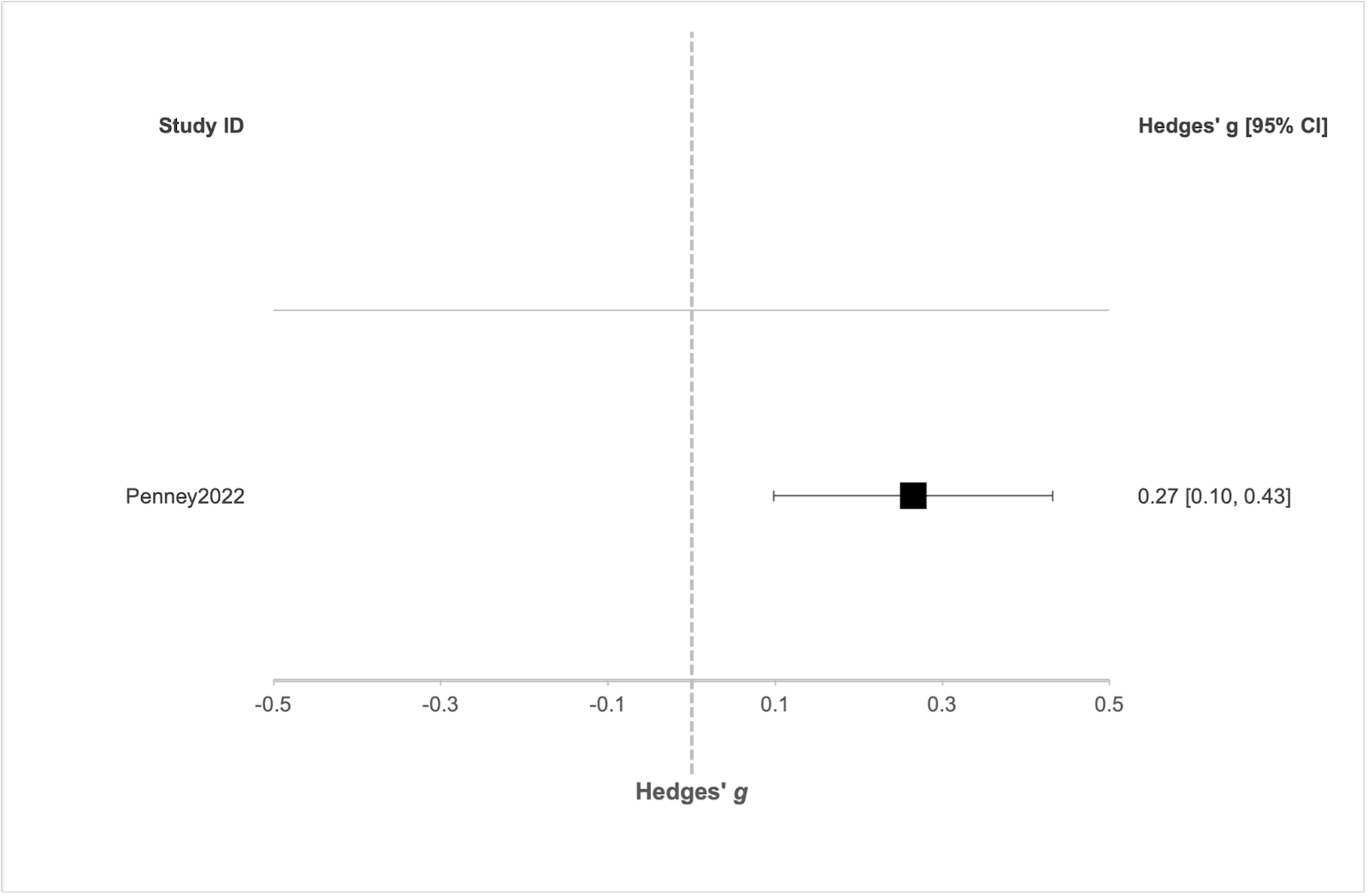


*Note*. Positive effect size values favor the intervention.

# eFigure 5. Forest Plot Depicting Effect Sizes for Negative Symptoms


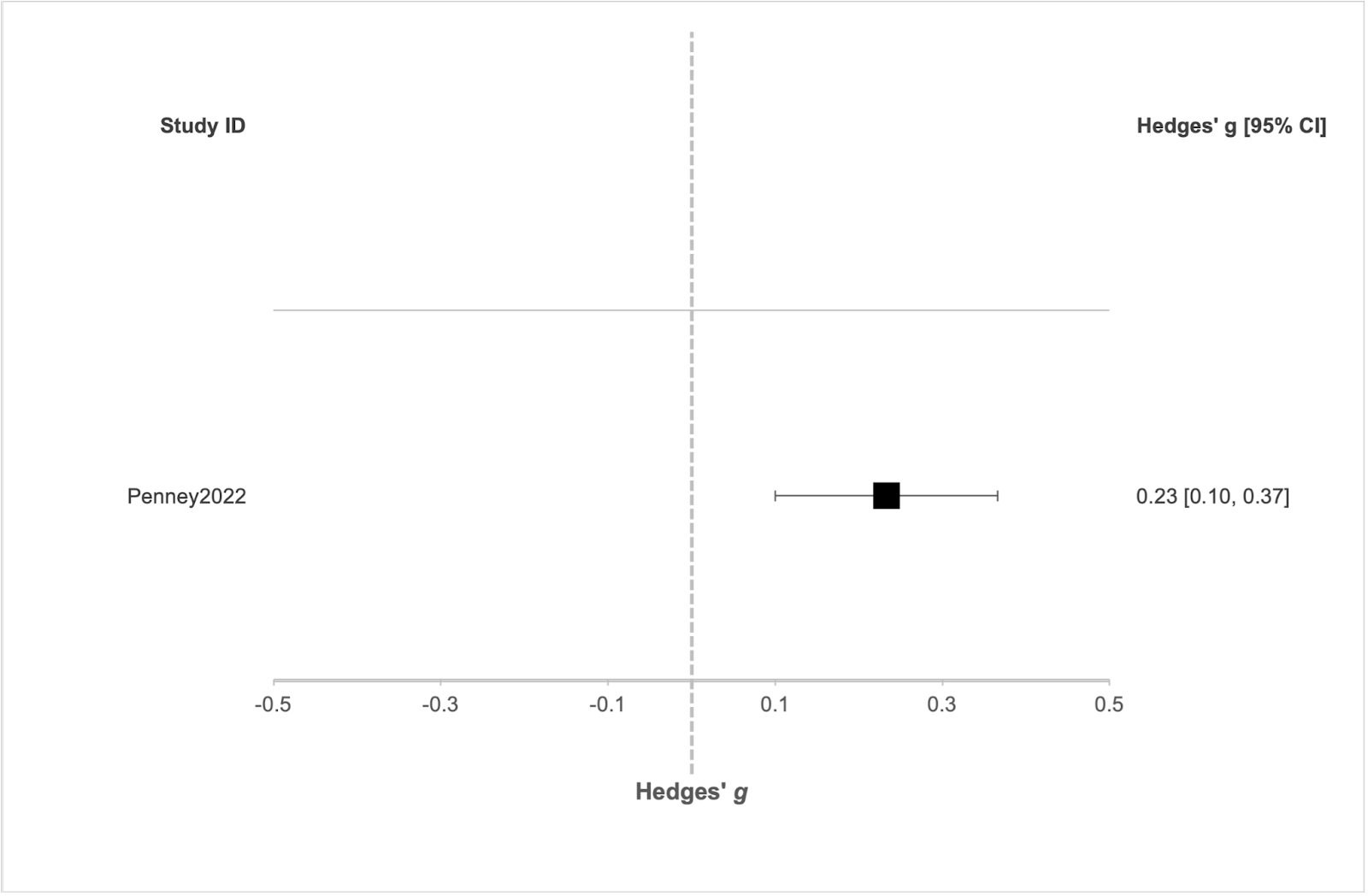


*Note*. Positive effect size values favor the intervention.

# eFigure 6. Histogram Depicting Effect Sizes in Favor of MCT for Delusions


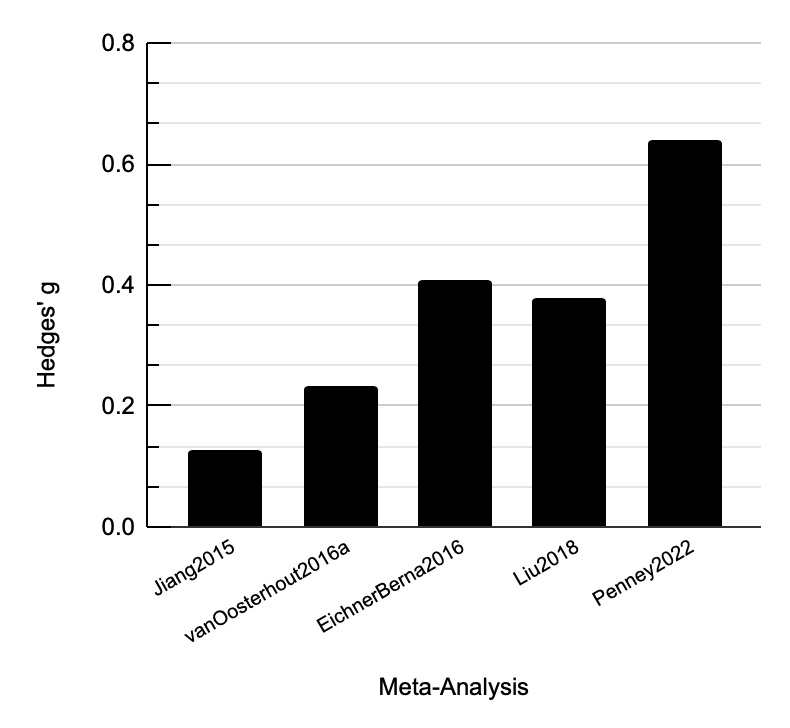


eFigure 7. Histogram Depicting Effect Sizes in Favor of MCT for Overall Symptoms


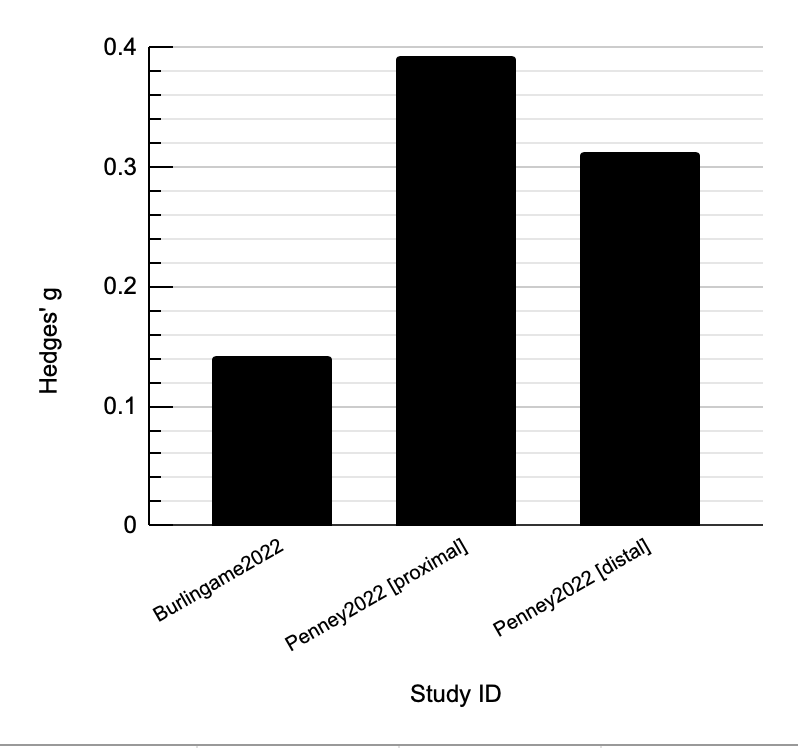


# eFigure 8. Histogram Depicting Effect Sizes in Favor of MCT for Positive Symptoms


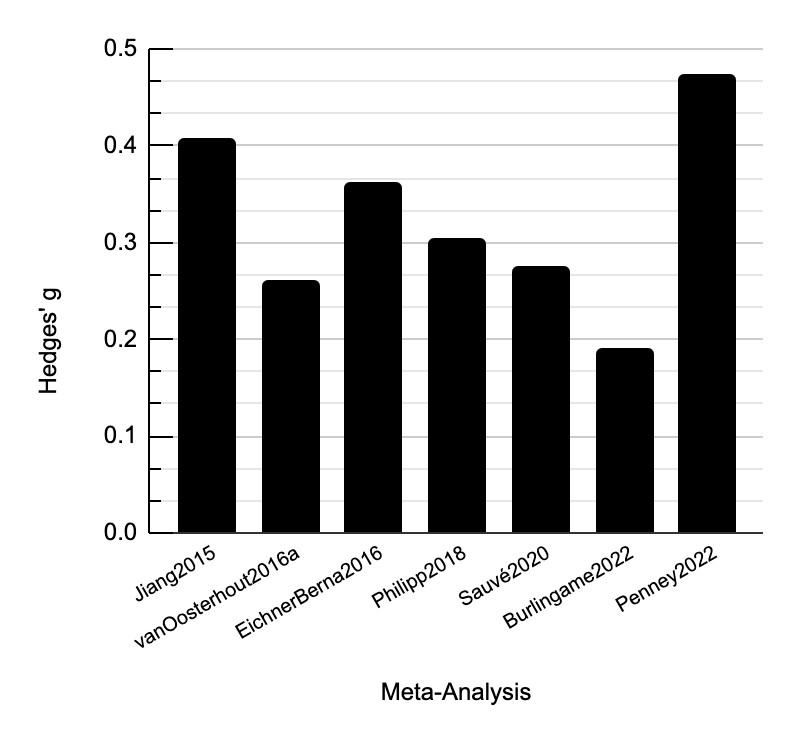


# eFigure 9. Histogram Depicting Effect Sizes in Favor of MCT for Hallucinations


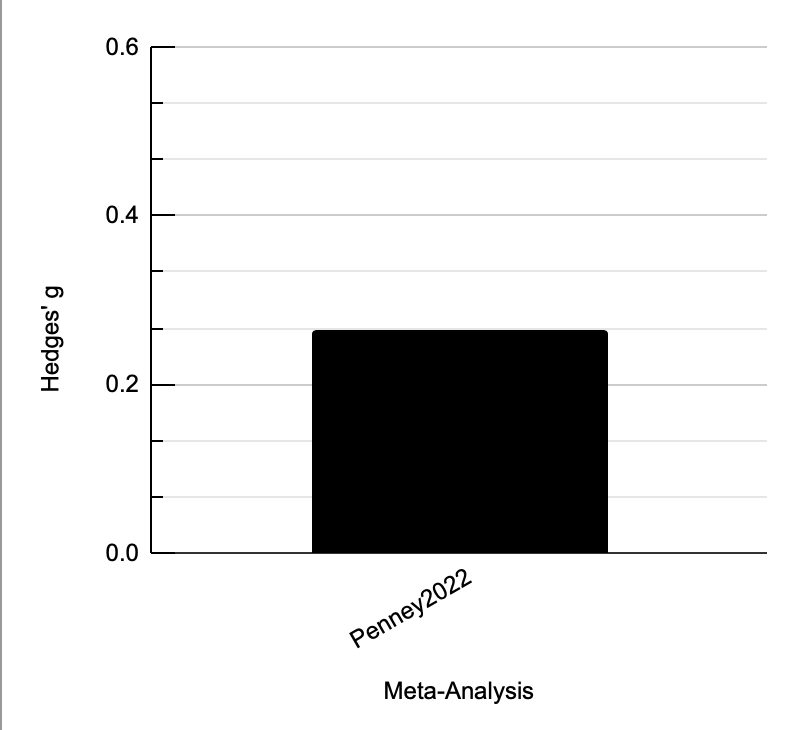


# eFigure 10. Histogram Depicting Effect Sizes in Favor of MCT for Negative Symptoms


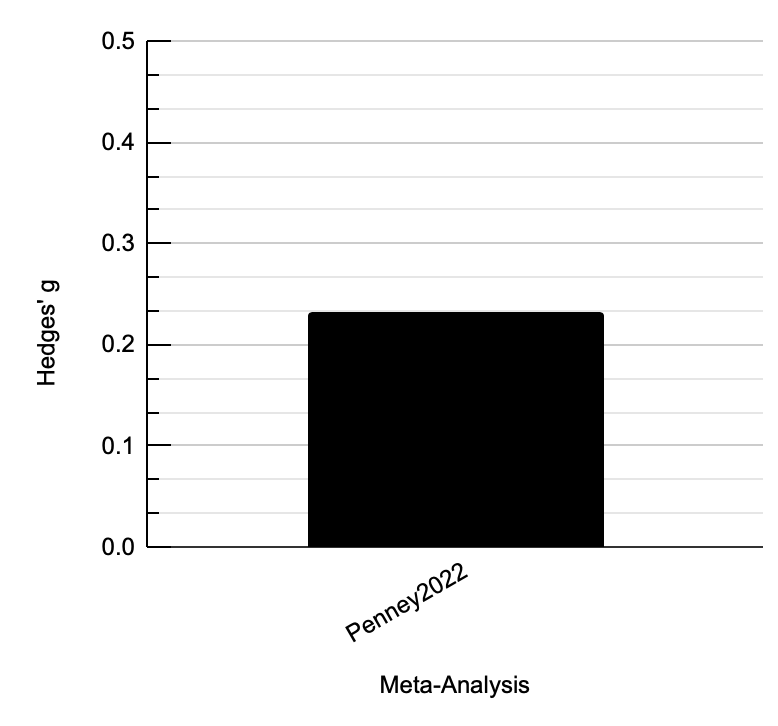

Supplement: Supplementary file 1 — Supplementary Material [file 41398_2025_3344_MOESM1_ESM.docx]
